# The Brazilian registry of kidney biopsies: results from the 2022 national prospective registry

**DOI:** 10.1590/2175-8239-JBN-2026-0096en

**Published:** 2026-10-02

**Authors:** Irene L. Noronha, Rodrigo José Ramalho, Claudia Maria Costa de Oliveira, Marilia Bahiense-Oliveira, Fabricio Augusto Marques Barbosa, Jose de Resende Barros, Ronny Mitsuoka, Osvaldo Merege Vieira-Neto, Precil Diego Miranda de Menezes Neves

**Affiliations:** 1 Sociedade Brasileira de Nefrologia, Departamento de Nefrologia Clínica, São Paulo, SP, Brazil.; 2 Universidade de São Paulo, Hospital das Clínicas, Faculdade de Medicina, Divisão de Nefrologia, São Paulo, SP, Brazil.; 3 Faculdade de Medicina de São José do Rio Preto, Divisão de Nefrologia, São Paulo, SP, Brazil.; 4 Hospital Universitário Walter Cantídio, Divisão de Nefrologia, Fortaleza, CE, Brazil.; 5 Universidade de São Paulo, Faculdade de Medicina de São Paulo, Hospital das Clínicas, Instituto de Radiologia, São Paulo, SP, Brazil.; 6 Universidade de São Paulo, Faculdade de Medicina de Ribeirão Preto, Departamento de Clínica Médica, Ribeirão Preto, SP, Brazil.

**Keywords:** Kidney Biopsy, Glomerular Diseases, Renal Pathology, Registry, Brazil

## Abstract

**Introduction:**

Kidney biopsy plays a central role in the etiological diagnosis and management of kidney diseases. The Brazilian Registry of Kidney Biopsies (BRKB) was established by the Brazilian Society of Nephrology to characterize biopsy-proven kidney diseases nationwide. The aim of this manuscript is to present the BRKB data for 2022.

**Methods:**

We analyzed data from adult patients who underwent native kidney biopsy and were prospectively included in the BRKB between January and December 2022. Demographic, clinical, laboratory, and histopathological variables were evaluated.

**Results:**

A total of 1,142 kidney biopsies from centers across all Brazilian regions were included. Female patients accounted for 54.8% of cases, and the mean age was 44.8 ± 16.4 years. The main indications for biopsy were kidney dysfunction (51.8%) and nephrotic syndrome (39.4%). Hypertension and diabetes mellitus were present in 47.8% and 15.1% of patients, respectively. At the time of biopsy, 67.1% had an estimated glomerular filtration rate < 60 mL/min/1.73 m^2^. Glomerular diseases were the most frequent histological diagnoses (72.5%), with lupus nephritis (23.1%), IgA nephropathy (15.0%), and focal segmental glomerulosclerosis (11.2%) being the most common.

**Conclusion:**

The 2022 BRKB data provide a contemporary overview of biopsy-proven kidney diseases in Brazil and highlight the predominance of glomerular disorders. Among diabetic patients undergoing biopsy, only half showed histological evidence of diabetic nephropathy, emphasizing the value of kidney biopsy in selected cases.

## INTRODUCTION

Chronic kidney disease (CKD) represents a major global public health challenge, affecting approximately 10–15% of the adult population worldwide and showing a sustained increase in prevalence over recent decades^1,2^. Hypertension and diabetes mellitus (DM) remain the leading causes of CKD globally; however, glomerular diseases continue to represent the second or third most frequent cause of kidney failure requiring kidney replacement therapy in many regions^3,4^.

Beyond their contribution to end-stage kidney disease, glomerular disorders are associated with substantial morbidity, heterogeneous clinical trajectories, and variable responses to therapy. In recent years, advances in immunopathology, molecular diagnostics, and targeted therapeutics have reshaped the field of glomerular diseases, reinforcing the importance of accurate etiological diagnosis and refined epidemiological characterization^5,6^. In this context, kidney biopsy remains the cornerstone for diagnosis, prognostic stratification, and therapeutic decision-making in patients with suspected glomerular disease.

In Brazil, national dialysis surveys consistently indicate that glomerular diseases account for approximately 5%–9% of patients receiving kidney replacement therapy, ranking among the leading non-diabetic causes of advanced CKD in the country^4^. Despite this relevance, comprehensive national data on biopsy-proven kidney diseases have historically been scarce. Most available evidence has been derived from single-center experiences or regional cohorts, limiting the ability to describe nationwide patterns, assess regional variability, or inform health policy planning^7,8,9,10,11^.

Earlier Brazilian studies, including large regional registries and institutional series, provided important insights into the local epidemiology of glomerular diseases but were restricted by geographic scope and temporal heterogeneity^7,8,9,10,11^. Moreover, changes in diagnostic practices, including the broader use of immunofluorescence, electron microscopy, and disease-specific biomarkers, have likely modified the observed spectrum of glomerular diseases over time, underscoring the need for contemporary, systematically collected data.

To address this gap, the Brazilian Society of Nephrology (SBN), through its Clinical Nephrology Department, established the Brazilian Registry of Kidney Biopsies (BRKB)^12^ as a national, multicenter, prospective registry designed to capture standardized clinical and histopathological data from native kidney biopsies performed across the country. The registry aims to provide a robust epidemiological framework for the characterization of kidney diseases in Brazil, with particular emphasis on glomerular disorders.

The first report of the BRKB described data from the initial year of platform implementation, including 954 biopsies performed in 2021, and demonstrated the feasibility of nationwide data collection^12^. Building on this foundation, the present study reports the results of the second year of the registry, encompassing kidney biopsies performed in 2022. By expanding the dataset and comparing findings across consecutive years, this analysis seeks to provide a more comprehensive and contemporary overview of the epidemiology of biopsy-proven kidney diseases in Brazil and to further establish the BRKB as a reference platform for clinical research and the development of health policy.

## METHODS

### Study design and registry structure

The BRKB is a national, multicenter, prospective, observational registry coordinated by the SBN through its Clinical Nephrology Department. The registry was designed to systematically collect standardized clinical and histopathological data from native kidney biopsies performed in adult patients across Brazil.

Data are entered into a secure, web-based electronic platform hosted by the SBN (https://www.glomerulopatias-sbn.org.br/inicio). The system is supported by a MySQL database with SSL-encrypted security protocols and does not store information that allows direct patient identification. To minimize duplicate records, the platform performs automated cross-checking based on patient initials, date of birth, and sex. Participating centers have access only to their own data, whereas centralized data management, extraction, and report generation are conducted by a data science professional affiliated with the SBN.

Participating nephrologists were recruited through the Clinical Nephrology Department of the SBN, which engaged representatives from the regional SBN branches and nephrologists with expertise in glomerular diseases across all five geographic regions of Brazil. Participation was voluntary and promoted through direct communication, presentations at national nephrology meetings, and SBN communication channels. Because kidney biopsies in Brazil are frequently interpreted by referral nephropathology laboratories serving multiple hospitals and nephrology practices, the exact number of participating centers could not be accurately determined using a conventional center-based framework. During the initial implementation phase, 45 university-based nephrology centers, together with additional private and non-university nephrology services, contributed biopsy cases to the registry.

This manuscript was prepared in accordance with Strengthening the Reporting of Observational Studies in Epidemiology (STROBE) guidelines for observational studies.

### Study population and inclusion criteria

The present analysis includes data from adult patients (≥ 18 years) who underwent native kidney biopsy between January 1 and December 31, 2022, and whose information was prospectively recorded in the BRKB. Pediatric biopsies and biopsies from kidney transplant recipients were not included. When more than one biopsy was performed on the same patient, only the first biopsy was considered for analysis.

Kidney biopsies were performed according to clinical indications established by the attending nephrologists at each participating center, reflecting routine clinical practice.

### Data collection and study variables

Clinical and demographic data were collected at the time of kidney biopsy using a standardized electronic questionnaire. Recorded variables included age, sex, self-reported race, biopsy date, the state where the procedure was performed, and region of origin. Race was self-reported by patients and recorded in the registry using predefined categories available in the electronic data capture system (White, Black, Brown/Pardo, Asian, or Indigenous).

Clinical indications for biopsy were categorized as nephrotic syndrome, non-nephrotic proteinuria, hematuria, rapidly progressive glomerulonephritis, or kidney dysfunction. Kidney dysfunction was recorded when impaired kidney function was considered by the treating nephrologist to be one of the indications for kidney biopsy. The registry did not distinguish between acute kidney injury, CKD progression, or other causes of renal function impairment. Information regarding comorbid conditions, including previously diagnosed hypertension and DM, as well as a personal or family history of kidney disease, was also collected. These variables were recorded as present, absent, or unknown. No centralized laboratory thresholds were established for these indications, reflecting the clinical assessment of the treating nephrologist.

Serum creatinine values at the time of biopsy were recorded, and the estimated glomerular filtration rate (eGFR) was automatically calculated by the platform using the CKD-EPI equation without race adjustment.

### Histopathological evaluation

Histopathological diagnoses were established by local nephropathologists based on light microscopy and immunofluorescence, with electron microscopy performed when available. The number of glomeruli present in each biopsy specimen was recorded. Interstitial fibrosis and tubular atrophy were assessed semiquantitatively and categorized as absent, mild (< 25%), moderate (25–50%), or severe (> 50%).

Immunofluorescence and/or immunohistochemistry analyses included evaluation for IgA, IgG, IgM, C3, C1q, kappa (κ) and lambda (λ) light chains, fibrinogen, and anti-PLA2R antibodies, with documentation of the location and intensity of deposits.

More than one histological diagnosis could be recorded for a given biopsy. In such cases, a primary histological diagnosis was defined based on a review by the coordinating committee, in consultation with the referring nephrologist and/or nephropathologist, and this diagnosis was used for epidemiological analyses.

### Classification of histological diagnoses

For analytical purposes, histological diagnoses were initially grouped into eight main categories: glomerular diseases, vascular diseases, tubulointerstitial diseases, monoclonal gammopathies, diabetic nephropathy, hereditary kidney diseases, miscellaneous diagnoses, and unclassifiable biopsies.

Detailed diagnostic options were provided within the electronic platform to ensure consistency of data entry across centers. Diagnoses were selected from predefined lists developed by members of the SBN Clinical Nephrology Department.

These broad diagnostic categories were adopted to facilitate epidemiological reporting and comparison of disease frequencies across the registry. It should be acknowledged that certain diseases may be classified into more than one pathological category; therefore, the classification was intended as a pragmatic framework for registry analyses rather than a strictly pathophysiological taxonomy.

### Statistical analysis

Continuous variables were described as mean ± standard deviation or median with interquartile range, according to data distribution. Categorical variables were expressed as absolute frequencies and percentages. All statistical analyses were performed using SPSS software (version 29.0, IBM Corp.).

### Ethical considerations

The BRKB study protocol was approved by the Ethics Committee of the *Faculdade de Medicina de São José do Rio Preto* (CAAE: 59947022.6.0000.5415; approval No. 5,586,556). The registry was approved as a national multicenter initiative coordinated by the SBN. The BRKB is a prospective observational registry, and the present study consisted of a retrospective analysis of anonymized data previously collected within the registry. Because the analyses were performed using de-identified registry data and involved no direct patient contact or intervention, the requirement for informed consent was waived by the Ethics Committee.

## RESULTS

### National representation of the cohort

In 2022, a total of 1,142 native kidney biopsies were included in the registry, with biopsies originating from centers across all five geographic regions of Brazil. The states with the highest number of registered biopsies were São Paulo, Minas Gerais, and Bahia. Most patients were from the Southeast region (63.9%), followed by the Northeast region (24.5%). The distribution of biopsies by state (absolute numbers) and by geographic region (proportions) is shown in Figure 1.

**Figure 1:**
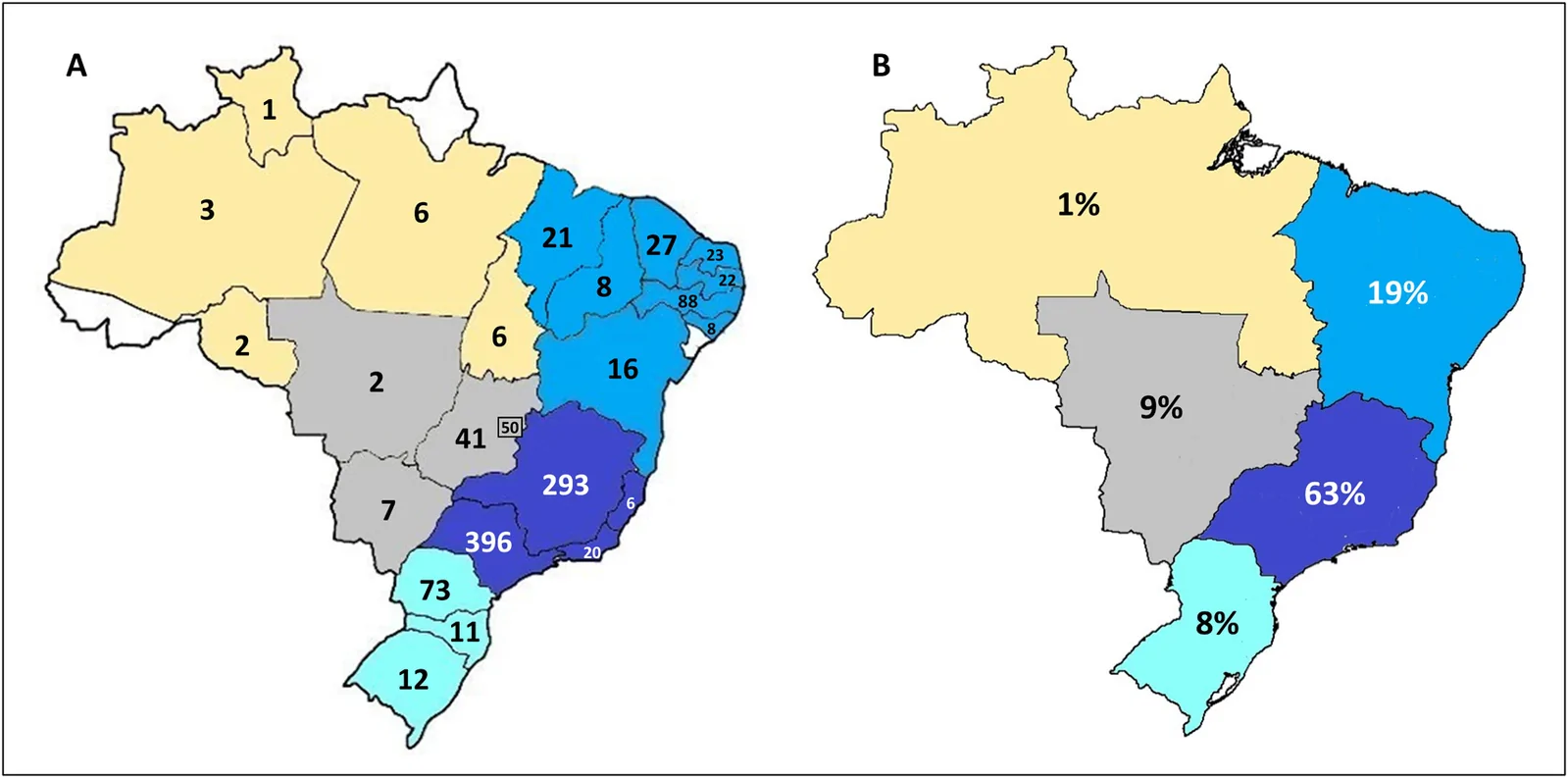
Geographic distribution of kidney biopsies included in the Brazilian Registry of Kidney Biopsies (BRKB) in 2022. (A) Total number of kidney biopsies by the state where the procedure was performed. (B) Proportion of biopsies across the five geographic regions of Brazil.

### Demographic characteristics

Mean age at biopsy was 44.8 ± 16.4 years, with the highest concentration in the 31–40-year age group (23%) (Figure 2). There was a slight predominance of female patients (54.8%). Regarding self-reported race, 61.7% of patients self-identified as mixed race (Pardo) and 32.5% as White; Black patients accounted for 5.0% and Asian patients for 0.8%. No Indigenous patients were recorded in 2022.

**Figure 2:**
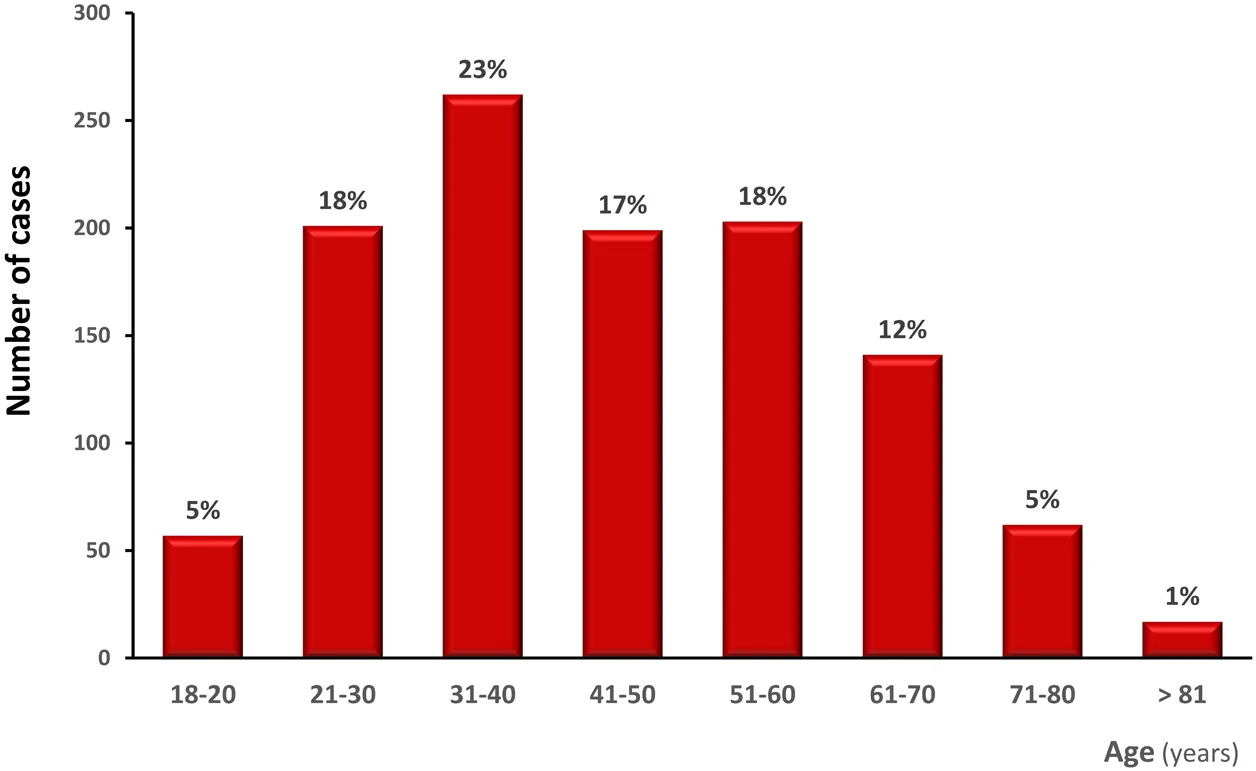
Distribution of kidney biopsies according to age group in the Brazilian Registry of Kidney Biopsies (BRKB) in 2022.

### Clinical and laboratory characteristics

The most common indication for kidney biopsy was kidney dysfunction (51.8%), followed by nephrotic syndrome (39.4%) (Figure 3). At the time of biopsy, 47.8% of patients had hypertension and 15.1% had DM. A family history of kidney disease was reported in 4.7% of cases. Kidney function was frequently impaired at the time of biopsy: 67.1% of patients had an eGFR <60 mL/min/1.73 m^2^, and 15.0% had eGFR <15 mL/min/1.73 m^2^ (Figure 4).

**Figure 3:**
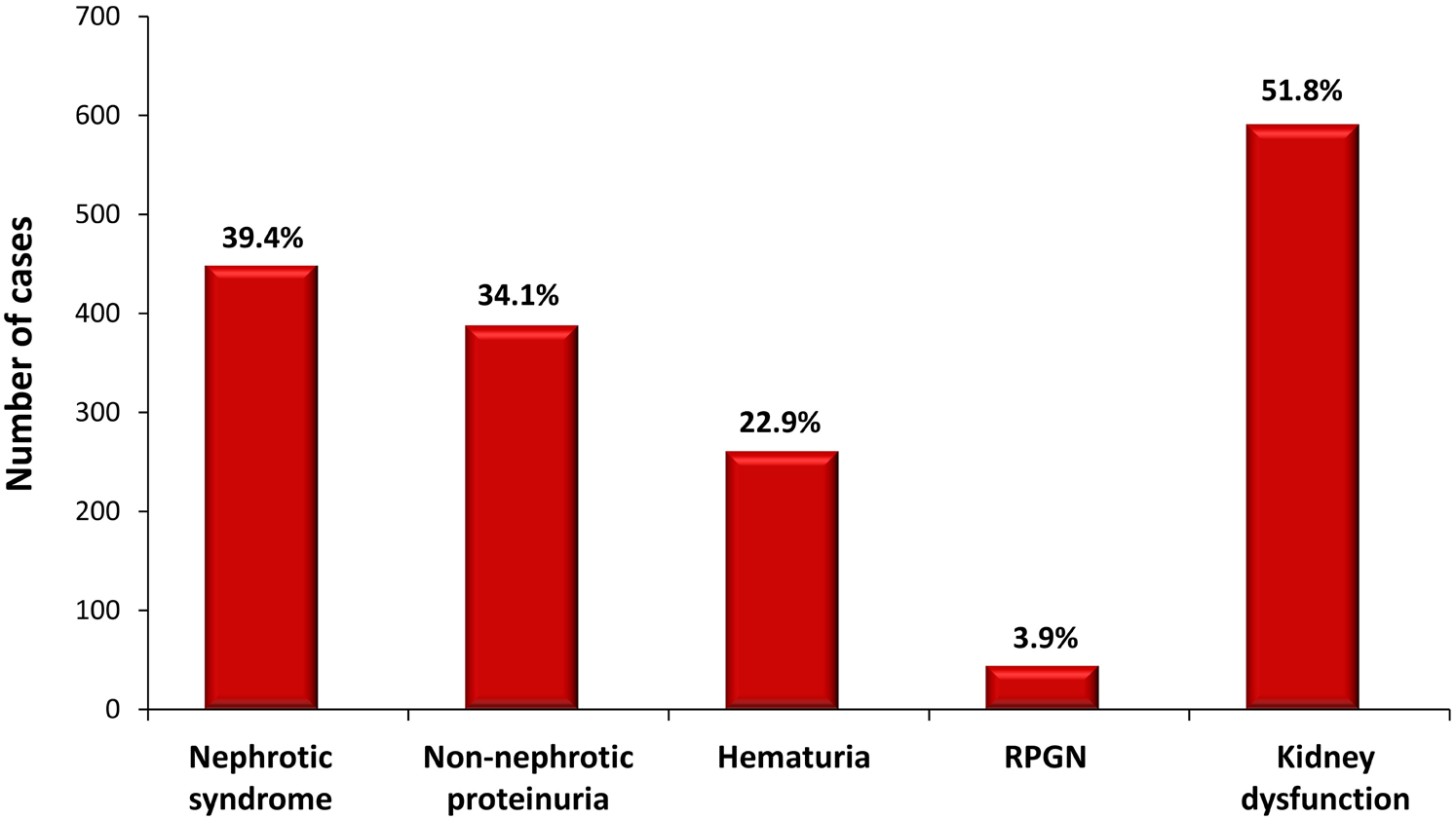
Distribution of clinical presentations at the time of kidney biopsy in the Brazilian Registry of Kidney Biopsies (BRKB) in 2022.

**Figure 4:**
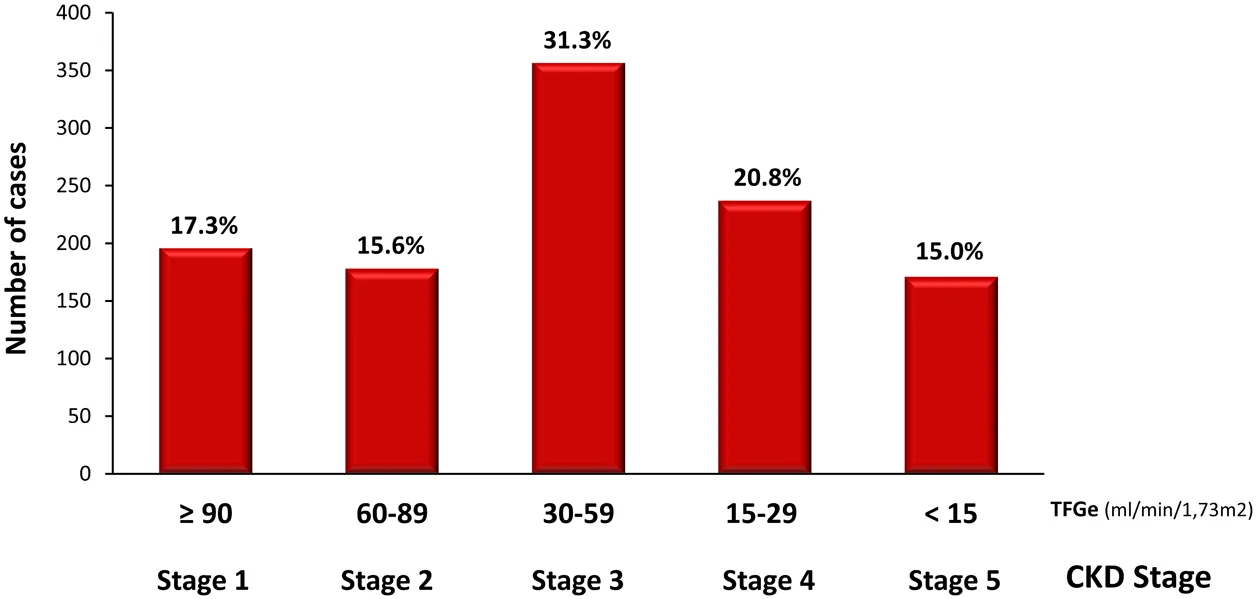
Distribution of patients according to estimated glomerular filtration rate (eGFR) and CKD stage at the time of kidney biopsy in the Brazilian Registry of Kidney Biopsies (BRKB) in 2022. Values above the bars indicate the percentage of all biopsies included in the registry.

### General characteristics of kidney biopsy samples

Most biopsy specimens showed adequate histological representation, with 78.5% containing ≥ 10 evaluable glomeruli (Figure 5A). Interstitial fibrosis and tubular atrophy were predominantly mild, observed in 60.2% of biopsies (Figure 5B). All cases underwent evaluation by immunofluorescence and/or immunohistochemistry. Electron microscopy was performed in only a subset of the cohort (6%).

**Figure 5:**
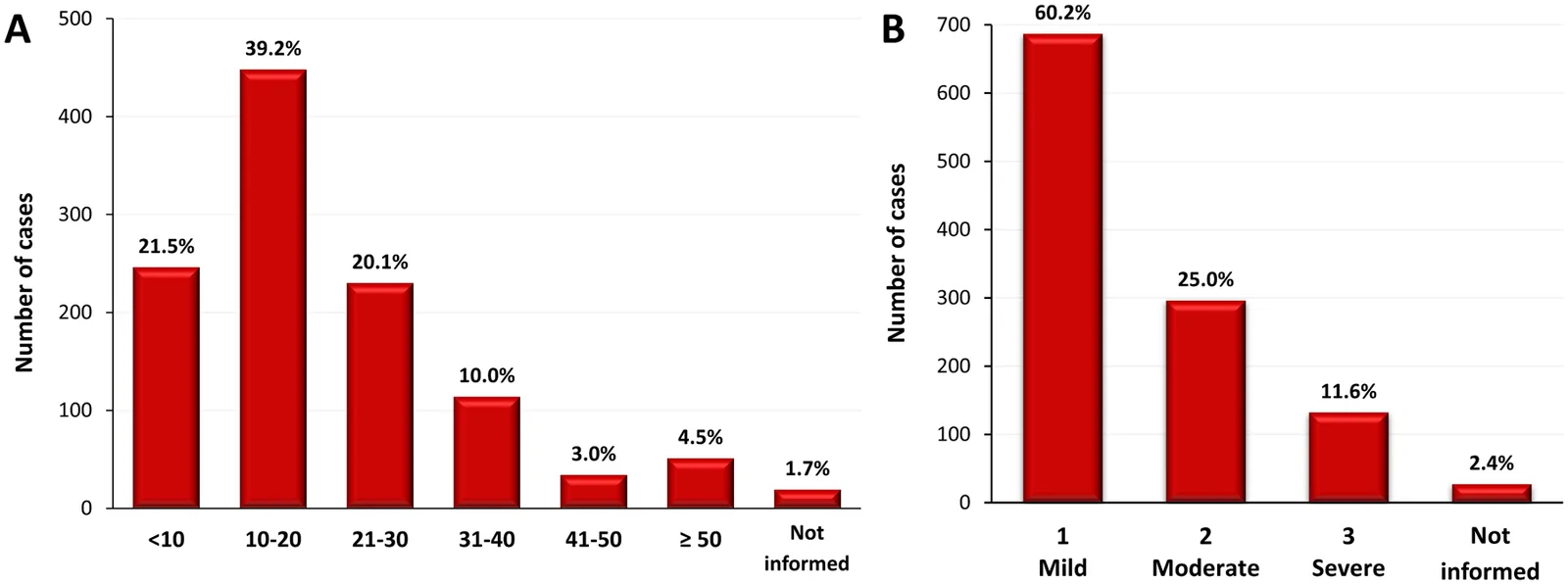
Histopathological characteristics of kidney biopsies included in the Brazilian Registry of Kidney Biopsies (BRKB) in 2022. (A) Number of glomeruli identified per biopsy specimen. (B) Degree of interstitial fibrosis and tubular atrophy (IFTA), categorized as mild (grade 1), moderate (grade 2), or severe (grade 3).

### Histological diagnoses

Glomerular diseases were the most frequent diagnostic category, accounting for 72.5% of all biopsies. The next most common categories were diabetic kidney disease (6.4%) and tubulointerstitial diseases (3.5%). In 11.0% of cases, a conclusive histological diagnosis could not be established (Figure 6). Most biopsies classified as “unclassifiable” did not reflect inadequate samples. Instead, they predominantly corresponded to advanced CKD characterized by extensive global glomerulosclerosis, tubular atrophy, interstitial fibrosis, and chronic irreversible parenchymal damage, precluding a more specific etiological diagnosis. A smaller proportion consisted of chronic tubulointerstitial nephritis with nonspecific features, chronic nonspecific renal lesions, or suboptimal biopsy samples.

**Figure 6:**
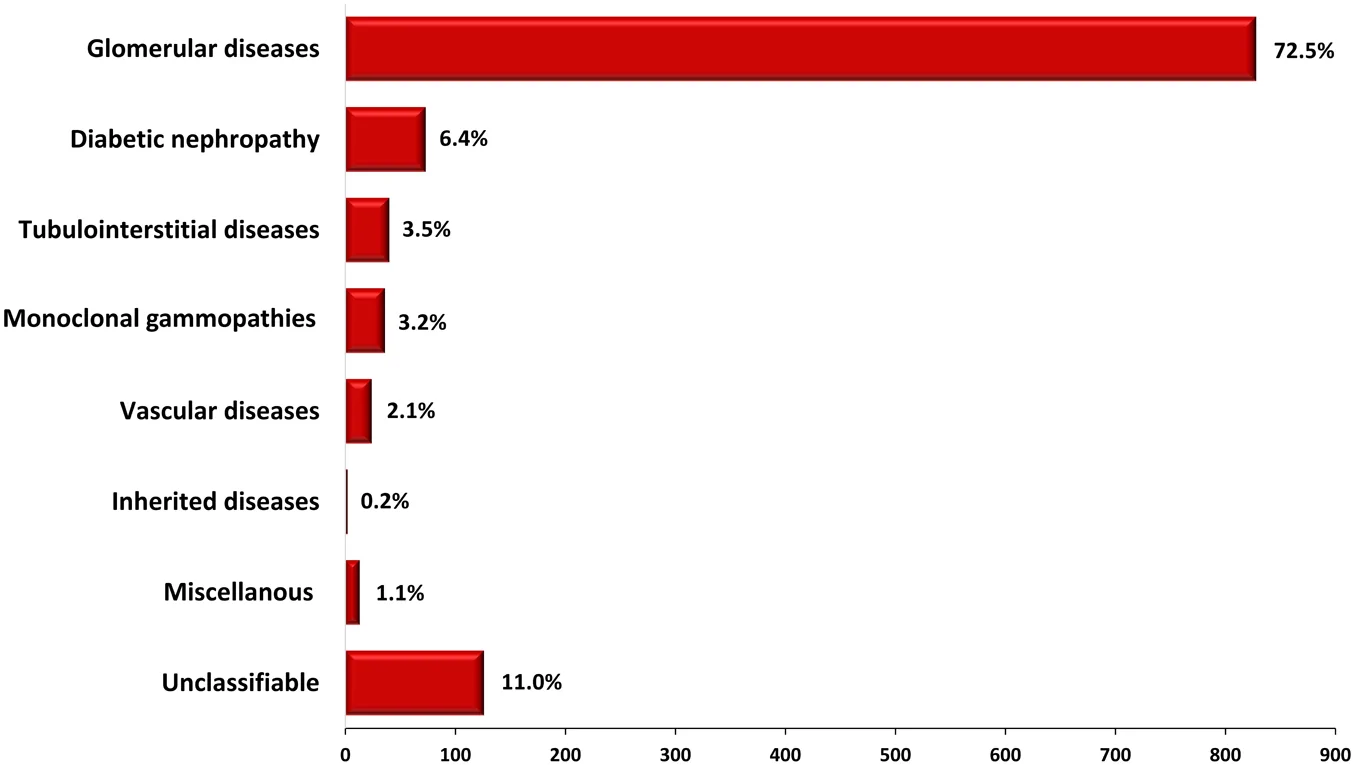
Distribution of histological diagnoses according to the major disease categories in the Brazilian Registry of Kidney Biopsies (BRKB) in 2022. For analytical purposes, histological diagnoses were grouped into eight major diagnostic categories. Values indicate the percentage of all biopsies included in the registry.

Among glomerular diseases, lupus nephritis (LN) was the most frequent diagnosis, accounting for 23.1% of cases, followed by IgA nephropathy (IgAN) (15.0%), focal segmental glomerulosclerosis (FSGS) (11.2%), and membranous nephropathy (MN) (9.5%) (Figure 7; Table 1).

**Figure 7:**
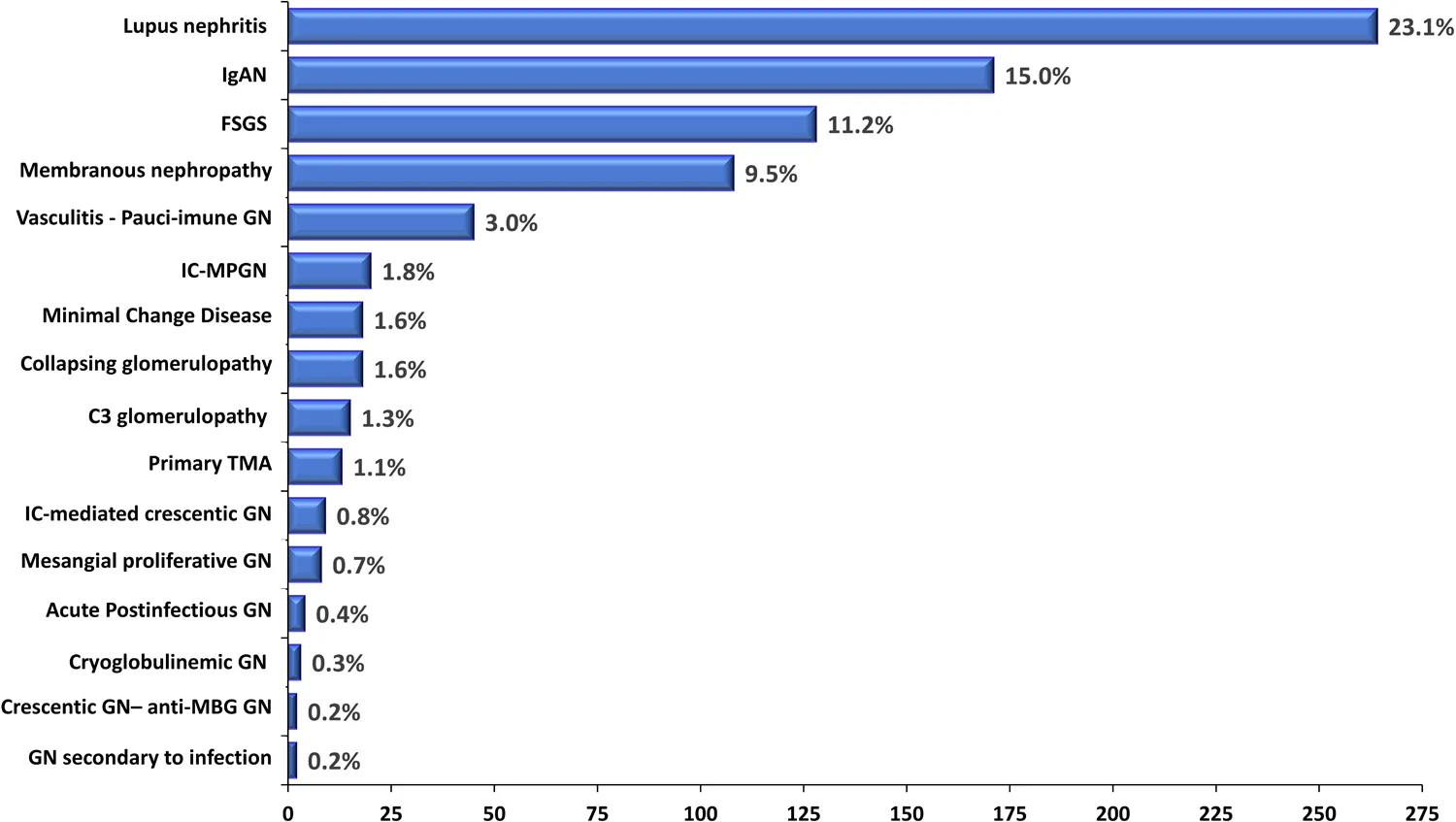
Spectrum and frequency of biopsy-proven glomerular diseases in the Brazilian Registry of Kidney Biopsies (BRKB) in 2022. The figure shows the distribution of histological diagnoses among biopsies classified as glomerular diseases. Percentages are expressed relative to the total number of biopsies included in the registry.

**Table 1:** Distribution of histological diagnoses in the Brazilian Registry of Kidney Biopsies (BRKB) in 2022, grouped according to the major diagnostic categories.

Glomerular diseases	n = 828	72.5%
Lupus nephritis	264	23.1%
IgA nephropathy	171	15.0%
Focal segmental glomerulosclerosis	128	11.2%
Membranous nephropathy	108	9.5%
Pauci-immune vasculitis	45	3.9%
Immune complex–mediated membranoproliferative GN	20	1.8%
Minimal change disease	18	1.6%
Collapsing glomerulopathy	18	1.6%
C3 glomerulopathy	15	1.3%
Thrombotic microangiopathy	13	1.1%
Immune complex–mediated crescentic GN	9	0.8%
Mesangial proliferative GN	8	0.7%
Acute postinfectious GN	4	0.4%
Cryoglobulinemic GN	3	0.3%
Anti-glomerular basement membrane GN	2	0.2%
Infection-related GN	2	0.2%
**Diabetic nephropathy**	**n = 73**	**6.4%**
**Tubulointerstitial diseases**	**n = 40**	**3.5%**
Acute interstitial nephritis	25	2.2%
Acute tubular necrosis	15	1.6%
**Monoclonal gammopathies**	**n = 36**	**3.2%**
Amyloidosis	17	1.5%
Light chain cast nephropathy	6	0.6%
Monoclonal immunoglobulin deposition disease	4	0.4%
Light chain proximal tubulopathy	1	0.1%
Other monoclonal gammopathies	3	0.3%
Proliferative glomerulonephritis with monoclonal immunoglobulin deposits	3	0.3%
Fibrillary glomerulonephritis	2	0.2%
**Vascular diseases**	**n = 24**	**2.1%**
Hypertensive nephrosclerosis	23	2.0%
Systemic sclerosis–associated kidney disease	1	0.1%
Hereditary kidney diseases	2	0.2%
Type IV collagen–related diseases	2	0.2%
**Miscellaneous**	**n = 13**	**1.1%**
**Unclassifiable**	**n = 126**	**11.0%**

Abbreviation – GN: glomerulonephritis.

Age stratification demonstrated higher frequencies of LN and FSGS among younger individuals. In the range of 31–50-year age group, LN and IgAN were the predominant diagnoses, whereas MN and diabetic nephropathy (DN) were more frequently observed in older age groups (Figure 8).

**Figure 8:**
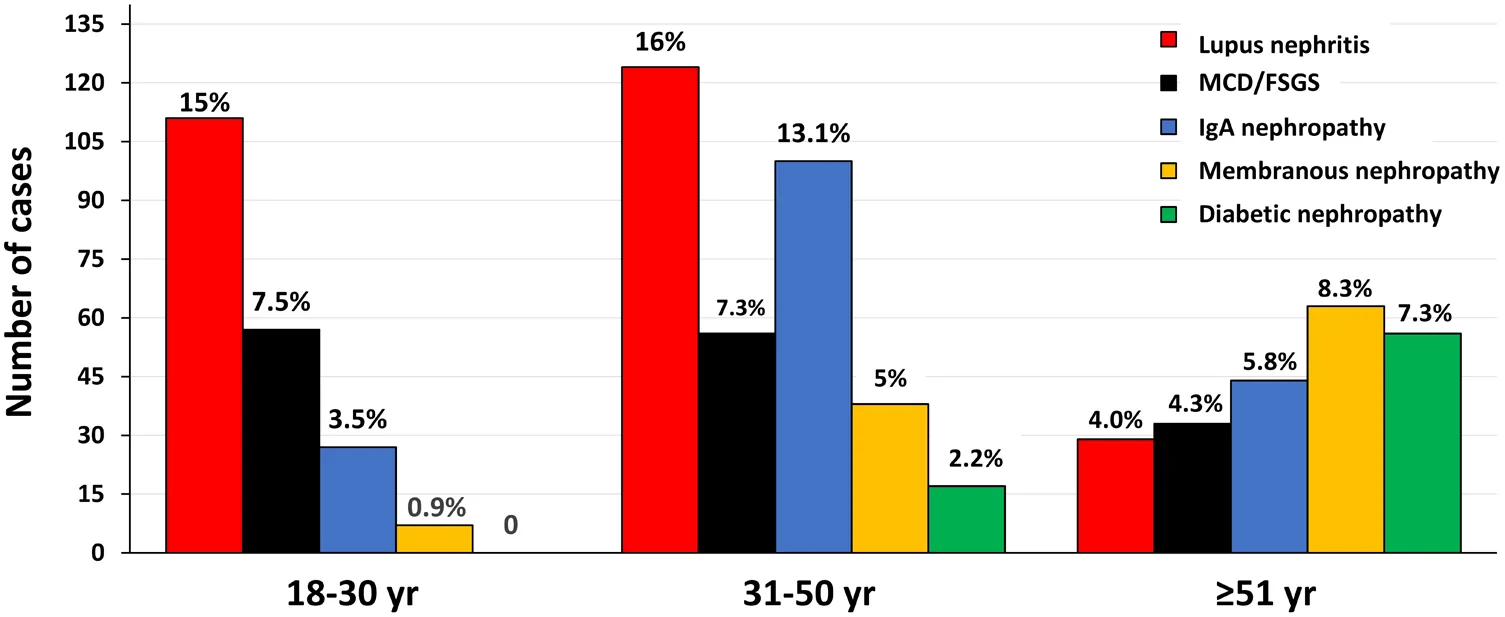
Age-stratified distribution of the most frequent biopsy-proven glomerular diseases in the Brazilian Registry of Kidney Biopsies (BRKB). The figure illustrates the distribution of lupus nephritis, FSGS/MCD, IgA nephropathy, membranous nephropathy, and diabetic nephropathy across three age groups (18–30 years, 31–50 years, and ≥ 51 years). Values above the bars indicate the percentage of all biopsies included in the registry.

Regarding sex distribution, LN, as expected, showed a marked predominance among female patients, whereas most other glomerular diseases were slightly more prevalent in males (Figure 9).

**Figure 9:**
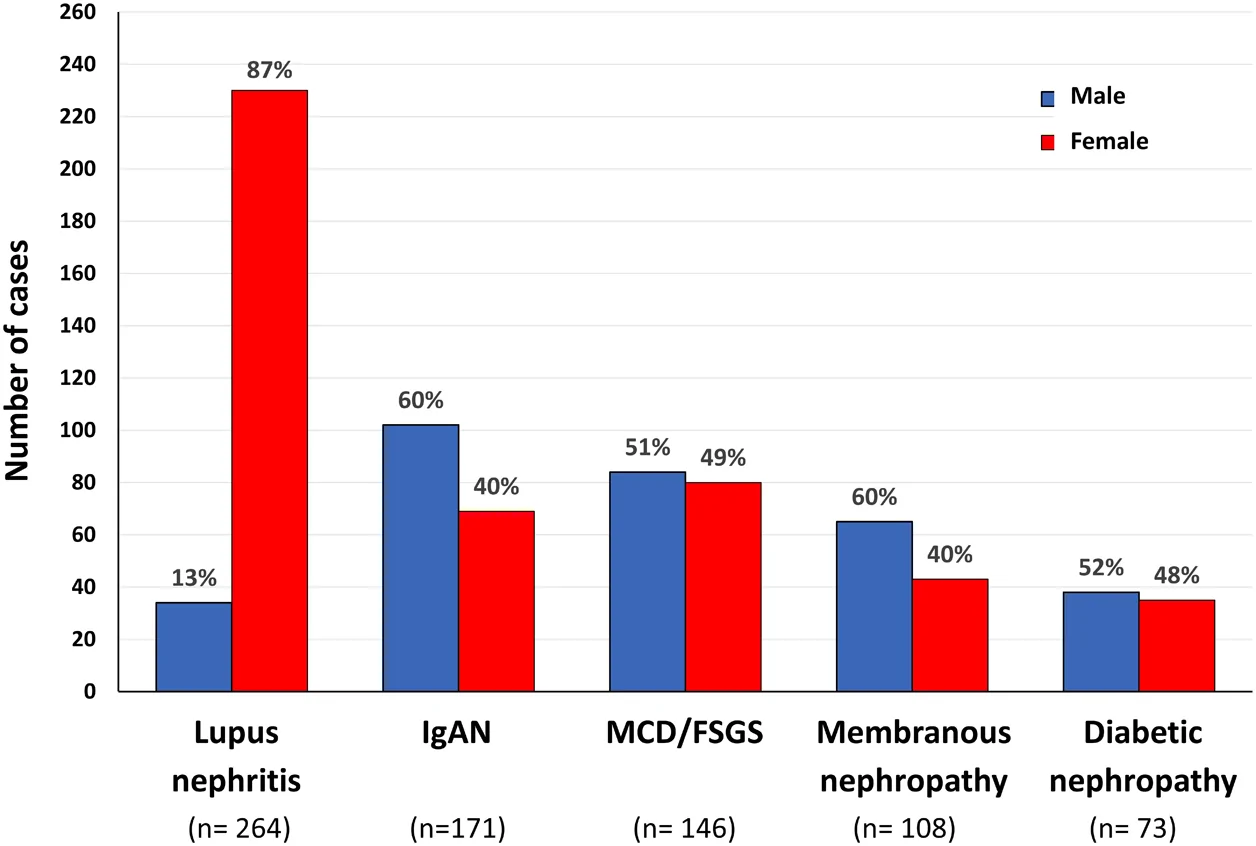
Sex distribution of the most frequent biopsy-proven glomerular diseases in the Brazilian Registry of Kidney Biopsies (BRKB). Values above the bars indicate the percentage of male and female patients within each diagnosis category. The total number of cases for each diagnosis is shown below.

Figure 10 depicts the clinical presentation at the time of kidney biopsy for the most common glomerulopathies in our registry. Nephrotic syndrome was the most frequent presentation among patients with focal segmental glomerulosclerosis/minimal change disease (FSGS/MCD) and MN and was also commonly observed in patients with DN. In contrast, non-nephrotic proteinuria and hematuria were more frequent among patients with LN and IgAN. Notably, with the exception of patients with FSGS/MCD, most patients in the other groups already had kidney dysfunction at the time of biopsy.

**Figure 10:**
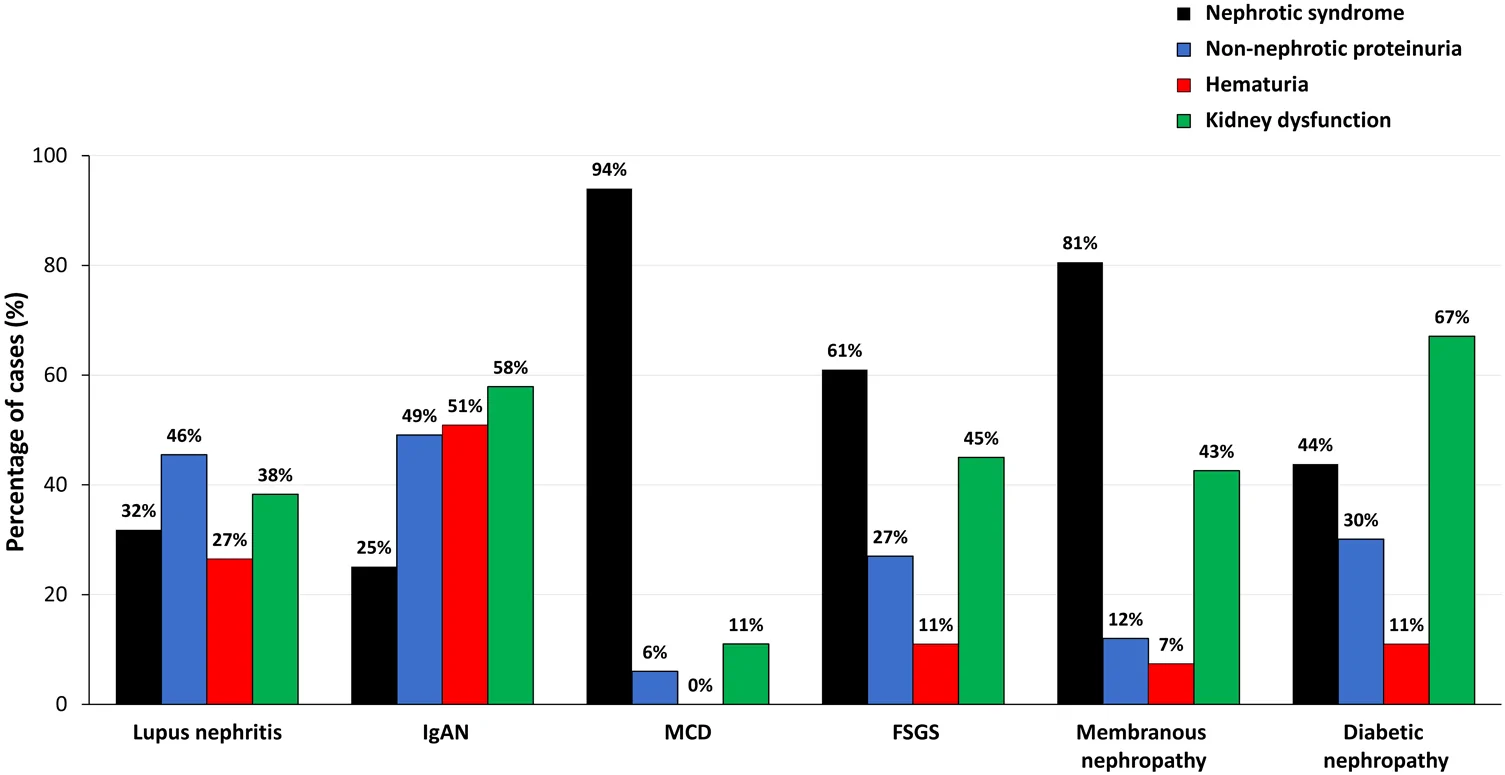
Frequency of clinical presentations characterized by nephrotic syndrome, non-nephrotic proteinuria, hematuria, or kidney dysfunction among patients with the most common biopsy-proven glomerular diseases. Values above the bars indicate the percentage of patients within each histological diagnosis category.

### Diabetic patients undergoing kidney biopsy

In the present cohort, 164 patients (14.4%) had a diagnosis of DM at the time of kidney biopsy (Figure 11). Notably, among these patients, only 82 (50.0%) showed histological features of DN, whereas the remaining 82 (50.0%) had no histological evidence of DN.

**Figure 11:**
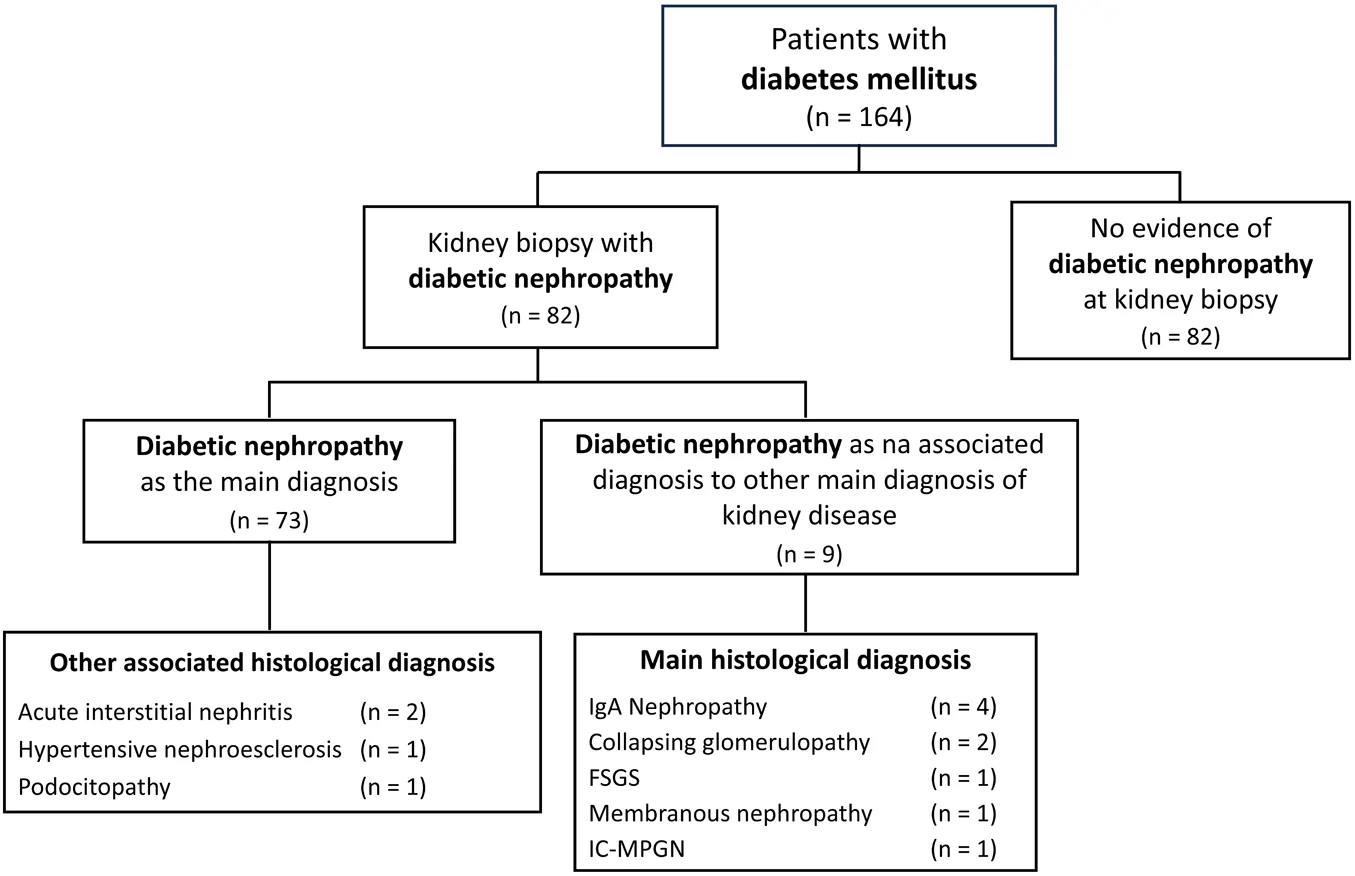
Histological findings in patients with diabetes mellitus who underwent kidney biopsy in the Brazilian Registry of Kidney Biopsies (BRKB) in 2022. Among 164 diabetic patients, 82 (50.0%) showed histological features of diabetic nephropathy, whereas 82 (50.0%) had no histological evidence of the disease. In 73 cases, diabetic nephropathy was the main histological diagnosis and, in 9, an associated histological diagnosis.

Among patients with DN, this condition was considered the primary histological diagnosis in 73 cases (89.0%), corresponding to 6.4% of all kidney biopsies included in the registry. In four of these patients (5.5%), additional histological findings were identified, including acute interstitial nephritis (n = 2), hypertensive nephrosclerosis (n = 1), and podocytopathy (n = 1). In the remaining 9 cases, DN was identified as an associated histological finding accompanying another primary kidney disease, including IgAN (n = 4), collapsing glomerulopathy (n = 2), FSGS (n = 1), MN (n = 1), and immune complex-mediated membranoproliferative glomerulonephritis (n = 1).

The mean age at biopsy was similar among diabetic patients with and without DN (57.9 ± 11.6 vs. 56.8 ± 12.2 years, respectively), as was the sex distribution. Patients with DN more frequently presented with impaired kidney function at the time of biopsy (67.1% vs. 39.9%). Nephrotic syndrome was the most common clinical presentation among patients with DN (46.3%), whereas hematuria was more frequently observed among diabetic patients without DN (19.5% vs. 12.2%).

Patients with DN also exhibited more severe impairment of kidney function at the time of biopsy. Median serum creatinine was significantly higher in patients with DN than in those without the condition (2.56 [IQR 1.72–4.11] vs. 1.63 [IQR 1.17–2.43] mg/dL; *p* = 0.0001), while median eGFR was significantly lower (24.9 [IQR 15.3–40.2] vs. 41.5 [IQR 24.7–62.7] mL/min/1.73 m^2^; *p* = 0.0003).

It is relevant to highlight that, compared with 2021, there were no reports of patients with HIV-associated nephropathies, IgG4-related kidney disease, immunotactoid glomerulonephritis, Fabry disease, or LCAT deficiency. Most are rare diseases and, for many of them, electron microscopy is essential for diagnosis. In this context, limited access to electron microscopy may represent a significant limitation for establishing these diagnoses.

## DISCUSSION

Reliable epidemiological data on glomerular diseases remain limited in many middle-income countries, despite the substantial burden of CKD and the widespread availability of kidney replacement therapies^2,13^. In Brazil, large national registries have successfully characterized dialysis and transplantation activity^4,14^; however, information derived from native kidney biopsies has historically been fragmented and largely restricted to single-center or regional series^7,15,16,17^. In this context, the BRKB represents a critical step toward systematically capturing biopsy-based data nationwide.

The present analysis, corresponding to the second year of the BRKB, reflects the transition of the registry from an initial implementation phase to a more consolidated data-collection platform^12^. In 2022, a substantial increase in the number of registered kidney biopsies was observed compared with the inaugural year, underscoring greater engagement of participating centers and improved incorporation of the registry into routine clinical practice. Similar maturation phases have been described in other national and regional biopsy registries. Importantly, this expansion occurred without major shifts in the geographic distribution of cases, with the Southeast region remaining predominant, followed by the Northeast. Rather than indicating sampling bias alone, this pattern likely mirrors the unequal distribution of nephrology services and referral centers across the country.

Beyond its quantitative growth, the 2022 BRKB dataset provides a more robust framework for describing the contemporary setting of kidney biopsy indications and histological diagnoses in Brazil. The stability of demographic characteristics and regional representation, combined with the increased sample size, strengthens the internal consistency of the registry and supports its use as a reference source for future epidemiological and clinical investigations. As the BRKB continues to expand, its longitudinal potential may allow more refined analyses of temporal trends, regional variability, and disease-specific outcomes, further enhancing its relevance for clinical decision-making and health policy planning.

The age distribution of patients undergoing kidney biopsy in 2022 closely mirrored that observed during the first year of the registry, with a predominance of young and middle-aged adults. Similar age profiles have been reported in biopsy registries from Latin America and Southern Europe^18^. The slight predominance of female patients differs from patterns reported in several international biopsy registries^19,20^ but is plausibly explained by the sustained high frequency of LN within the Brazilian cohort. When comparing the 2022 dataset with the 2021 BRKB report, numerous relevant observations emerge. First, there was a substantial increase in the number of registered biopsies, reflecting the progressive consolidation of the registry and broader participation of centers. Despite this expansion, demographic characteristics, clinical indications, and the overall distribution of major diagnostic categories remained remarkably stable, supporting the internal consistency of the platform.

Self-reported race in Brazil reflects a highly mixed-race population, shaped by diverse ancestral contributions^21,22^. In the present cohort, a high proportion of patients self-identified as mixed race (Pardo), exceeding the proportion reported in the first year of the registry. Rather than implying disease-specific racial clustering, this finding likely reflects broader geographic participation and improved completeness of demographic data in 2022. These observations reinforce the limited utility of self-reported race as a surrogate for genetic ancestry in epidemiological studies of kidney disease in Brazil^22,23^. Notably, the proportion of biopsies classified as “unclassifiable” increased in 2022. A detailed review of these cases demonstrated that most did not result from inadequate biopsy samples or diagnostic disagreement among nephropathologists. Rather, the majority corresponded to advanced CKD with extensive global glomerulosclerosis, tubular atrophy, interstitial fibrosis, and irreversible chronic parenchymal damage, which precluded the identification of a specific underlying etiology. A smaller proportion consisted of chronic tubulointerstitial nephritis with nonspecific features or lesions considered insufficiently characteristic to support a definitive diagnosis. Nevertheless, limited access to electron microscopy may also have contributed to diagnostic uncertainty in a subset of cases, particularly in diseases in which ultrastructural evaluation is important for establishing a definitive diagnosis or refining histopathological classification.

Regarding clinical indications, kidney dysfunction remained the leading reason for performing a native kidney biopsy, followed by nephrotic syndrome, reproducing the overall pattern observed in 2021 and in other large biopsy registries^24,25^. Notably, more than two-thirds of patients had an eGFR below 60 mL/min/1.73 m^2^ at the time of biopsy, indicating that a substantial proportion of procedures were performed in individuals with established CKD. Similar findings have been reported in contemporary cohorts, reflecting a tendency toward delayed biopsy in routine clinical practice^26^. Despite this, the predominance of mild interstitial fibrosis and tubular atrophy suggests that many patients were biopsied at stages where diagnostic clarification could still influence management and potentially modify the disease trajectory. These findings underscore the continued relevance of kidney biopsy in patients with reduced kidney function, particularly when the clinical presentation is atypical or diagnostic uncertainty persists^27,28^.

In terms of disease spectrum, LN remained the most frequent diagnosis across both years. A modest increase in the relative frequency of IgAN was observed in 2022, further reinforcing its epidemiological relevance in Brazil and possibly reflecting improved recognition of the disease as registry coverage expands. Importantly, the relative frequencies of other major glomerular diagnoses, including FSGS and MN, remained largely stable, suggesting the preservation of the overall diagnostic profile despite the expansion of the registry.

In the second year of the BRKB, glomerular diseases continued to account for most histological diagnoses, representing nearly three quarters of all biopsies performed. This pattern is consistent with that observed in most national and international biopsy registries^3,4,29^. The persistence of this distribution underscores the central role of glomerular disorders as the primary indication for native kidney biopsy in Brazil. At the same time, the higher proportion of biopsies classified as inconclusive highlights ongoing diagnostic challenges, which may be related to differences in case complexity, the availability of ancillary techniques, or variability in local diagnostic workflows across participating centers^24^.

Within the spectrum of glomerular diseases, LN remained the most frequent diagnosis, followed by IgAN and FSGS. This distribution resembles that reported in several Latin American cohorts but differs from patterns observed in East Asia and parts of Europe, where IgAN predominates^30,31^. The increased incidence of IgAN in 2022 further supports its relevance as a major cause of glomerular disease in Brazil, consistent with the broader recognition of this entity as diagnostic capabilities improve and registry coverage broadens^6^.

Sex- and age-related patterns observed in 2022 were consistent with the biological behavior of the underlying diseases. LN predominated among women, whereas other glomerular diseases were more frequently diagnosed in men. Age-stratified analyses revealed distinct patterns across the major glomerular diseases. LN predominated among younger adults, particularly between 18 and 50 years, reflecting the known epidemiology of systemic lupus erythematosus. In contrast, MN and DN were substantially more frequent among older individuals, particularly those older than 50 years. IgAN showed a peak frequency in middle-aged adults, whereas FSGS and MCD were distributed across all age groups. These findings are broadly consistent with international biopsy registries and support the biological plausibility of the BRKB dataset^18,32,33,34^.

Although diabetic kidney disease represented a minority of histological diagnoses, its contribution remains clinically significant. Notably, almost half of the patients with diabetes who underwent kidney biopsy did not exhibit histological features compatible with DN, and a substantial proportion presented with superimposed or alternative diagnoses, most commonly IgAN. Similar observations have been consistently reported in biopsy-based studies of diabetic patients^35,36,37^. These findings underscore the continued importance of kidney biopsy in selected patients with diabetes, particularly when the clinical presentation is atypical or the disease course deviates from the expected pattern^38^.

Importantly, diabetic patients undergoing kidney biopsy do not represent the overall population of individuals with DM. In routine clinical practice, kidney biopsy is usually reserved for patients with atypical clinical presentations, rapid deterioration of kidney function, active urinary sediment, nephrotic syndrome, or suspicion of superimposed kidney disease. Therefore, the high proportion of non-diabetic kidney diseases and mixed histological patterns observed in the present cohort should be interpreted within the context of a selected biopsy population rather than as representative of the overall population of diabetic patients. Nevertheless, these findings reinforce the diagnostic value of kidney biopsy in selected diabetic patients and highlight the limitations of relying exclusively on clinical criteria to diagnose diabetic kidney disease.

An additional consideration relates to the organization of kidney biopsy services in Brazil. Unlike dialysis registries, kidney biopsies are frequently processed in referral nephropathology laboratories that receive specimens from multiple hospitals and nephrology practices. Consequently, the number of participating centers cannot be precisely defined using a conventional center-based approach. Although participation was voluntary and regional contributions were uneven, biopsy cases were obtained from all five geographic regions of Brazil, supporting the nationwide scope of the registry while acknowledging that the predominance of the Southeast region may influence its external validity.

Some limitations of this study should be acknowledged. Participation in the registry remains voluntary, with unequal representation across regions, and the absence of longitudinal follow-up currently limits prognostic analyses. In addition, variability in access to advanced diagnostic tools, such as electron microscopy and molecular techniques, may influence diagnostic classification in a subset of cases^39,40,41^. Nevertheless, the BRKB constitutes the most comprehensive national effort to date to systematically characterize findings from kidney biopsy in Brazil.

In conclusion, the 2022 data consolidate the BRKB as a robust and internally consistent national registry. The stability of diagnostic patterns across consecutive years, coupled with a growing number of registered biopsies, supports the reliability of the platform and its potential for future analyses. Continued expansion of the registry, together with the incorporation of longitudinal clinical data, may provide a valuable foundation for epidemiological research, health policy planning, and the rational implementation of emerging therapies in Brazilian nephrology, as well as for supporting the development of a more integrated national renal pathology network across the country.

## Data Availability

The datasets generated and/or analyzed during the study are available from the Brazilian Society of Nephrology upon reasonable request.

## SUPPLEMENTARY MATERIAL

The following online material is available for this article:

Supplementary Material - List of collaborators.
