# Supplementary material for: The Brazilian registry of kidney biopsies: results from the 2022 national prospective registry
Source: J Bras Nefrol. 2026 Oct 2;48(4):e20260096. doi: 10.1590/2175-8239-JBN-2026-0096en (PMC13645486; doi:10.1590/2175-8239-JBN-2026-0096en)
Supplement: Supplementary file 1 [file 2175-8239-jbn-48-4-e20260096-20260728-suppl1.pdf]

Braz. J. Nephrol. 2026, 48(4): e20260096

https://doi.org/10.1590/2175-8239-JBN-2026-0096en

Supplementary Material to "The Brazilian registry of kidney biopsies: results from the 2022 national prospective registry"

List of collaborators

Adalberto F Nogueira, Ademilson A Feitosa, Adriana A De A Melo, Adriana Bittencourt, Adriana L Gomes, Adriana R V De Oliveira, Adriano D Marques, Adriel G Da Silva, Alaour C Duarte, Alcino R Mendes, Alessandra C De Oliveira, Alessandra J De C T L Leite, Alexandre C P Coelho, Aleysson F Terra, Alice G S Aquino, Alice P Naseri, Aline M Rodrigues, Aline N P Luz, Aline O Menezes, Alisson D Maciel, Alisson L J Oliveira, Amanda T De Melo, Ana C N Tome, Ana C Tome, Ana C A Cardoso, Ana C P De F Fontes, Ana L L De C R Cabeça, Andre A De Oliveira, Andre C A De Lima, Andre M Faria, Andrea C De A P Machado, Andreia Queiroz, Angelo E De F Paschoalini, Anna L B Lima, Antonio E V Junior, Antonio F Coutinho, Antonio F Coutinho Filho, Aparecida P G Visona, Ariel A De B Rosa, Arnaldo M Neto, Arthur A A Venturini, Austen Ir M Coelho, Ayrla P B Lira, Barbara D Jones, Barbara L Peralva, Barbara M V C Silva, Barbara P Martins, Barbara Santana, Beatriz S De Carvalho, Bernardo R Antonini, Boris A Cruz, Braulio H V De Figueiredo, Brenda C O Marquess, Breno A C Soares, Bruna A M Do Nascimento, Bruna R Moreira, Bruna S De Oliveira, Bruna T Alves, Bruno A Silva, Bruno T Gomes, Camila Barbosa, Camila M Lopes, Camila R L Garcia, Carlos A A Bornancin, Carlos D Gomes, Carlos E M Rocha, Cassiana M Fraga, Cassiano A B Silva, Cecilia T Peixoto, Celso Fianco, Cezar G De Carvalho, Ciro B S Costa, Clara A P Leao, Claudia L S Neiva, Claudia M C De Oliveira, Claus D Dummer, Criselen D Sinete, Cristela M Ceretta, Cynthia R De Oliveira, Daniel Monteiro, Daniela M Oliveira, Daniela P M De Souza, Daniela S Freitas, Daniele O Ginar, David C Wanderley, David J B Machado, Dayane Elias, Debora M Casassanta, Denio B De Souza, Denise Costa, Denise L

X Tavares, Diego C Eleuterio, Diego D A Polido, Diego S Rodrigues, Divino U Mendonca, Domingos C Chula, Horacio J Ramalho, Francisco Rasiah, Vito B Neto, Edmundo O Raspanti, Eduardo A Tavares, Eduardo B G V Batista, Eduardo G Mattar, Eliana M P Fortes, Elias B De S Queiroz, Elida Carvalho, Eline Nogueira, Elisandro V Rodrigues, Emilio H B Maciel, Epitacio R D L Neto, Erika N H Lee, Estefane L M Vasconcelos, Estevam A Viotti, Evaldo G Terra, Fabiano B Custodio, Fabiano F M Prado, Fabiano M D Neves, Fabio Auriemma, Fabio H Ferraz, Fabio M Ogata, Fabio Reis, Fabiola P Bremer, Fabricio A M Barbosa, Fabricio P Gusmao, Fatima C M Pelarizo, Felipe M Tome, Fernado Sales, Fernanda Camelo, Fernanda C C Sanches, Fernanda M Ribeiro, Fernanda Q M Marques, Fernanda S G Polachini, Fernando A Vita, Fernando D M De L Junior, Fernando Sales, Flavia M Ferreira, Flavio M Pinto, Flavio R D Aguiar, Franklin C Barcellos, Fransico Rasiah, Frederico C B Cavalcanti, Frederico Guarino, Gabriel P H Silveira, Gabriela L L Moreira, Gabriela P Coral, Geraldo Amorim, Geraldo M M De Paula, Gildasio Rocha, Gisele De Souza, Gisele V Fernandes, Gloria Y S A Guzman, Guilherme C Antunes, Guilherme D R Raposo, Gustavo E Neves, Gustavo J Sebba, Gustavo Marinelli, Gustavo N Betonico, Gustavo V Araujo, Gyl E B Silva, Hanna K A G Machado, Heitor A Rocha, Helen S S Cardoso, Heleno B De Oliveira, Heloisa R Vianna, Henrique N Watanabe, Horacio J Ramalho, Iara M Cunha, Iara S P L Silva, Igor C Almeida, Igor T Alvim, Igor T A Cardoso, Isabela B Pinto, Isabela F Romualdo, Isabella C Do Carmo, Italo Alves, Italo R C Alves, Ivan D M Araujo, Jair D Junior, Jassonio Mendonca, Joao B Z Moreira, Joao F Picollo, Joao F P De Oliveira, Joao P Zumack, Jonathan J D A Rabelo, Jorge P O Felliciano, Jose A P Junior, Jose D S M Neto, Jose D R B Neto, Julia D P De Morais, Juliana B C Tassi, Juliana D C Matos, Juliana E G Leme, Juliana F P Miguel, Juliana G Bordon, Juliana M De Carvalho, Julio C S Barreto, Kalil G L Coelho, Katia A Ramos, Kellen M A H Costa, Kildere Moura, Ladislau J F Junior, Larissa A Miranda, Larissa A Pereira, Larissa D A Santos, Larissa G D F Andrade, Lauro V M Da Silva, Leandra R C De Carvalho, Leandro D C B A Soares, Leandro S Miranda, Leandro Vasconcelos, Leonardo A Pacini, Leonardo A C Pereira, Leonardo Verona, Livia B Cavalcante, Lizia R C Ribeiro, Loise L C Turra, Lorena F Maia, Luana C S Da Silva, Lucas V De Assis, Luciana Batista, Luciana K C Batista, Luciana Percegona, Luciano F Coelho, Lucio Silva, Luiz C Pavanetti, Luiz F C Giordano,

Luiz Moura, Luiza R Rezende, Luzia O Braga, Maira C Z Arouca, Maira I C Santos, Marcela O F B Borges, Marcella S Nessralla, Marcella S Laferreira, Marcelo D P Bianco, Marcelo M Rodrigues, Marcelo P M Filho, Marcelo R Bastos, Marcia H F G De Abreu, Marcia M Dias, Marcia R Santos, Marclebio M C Dourado, Marcus F Lasmar, Maria A S F B Baptista, Maria C B Moreira, Maria C Da Silva, Maria D F C R De Melo, Maria G Polito, Maria J C C Brandao, Mariana G P De Paula, Mariana P Veloso, Mariana S Braga, Marilda S L Veloso, Marilia Q Neves, Marilinson B Rosario, Marinella P D A Ananias, Mario H De O Junior, Mario L Luvizotto, Maristela Bohlke, Marlene A Dos Reis, Michel N Zigamantas, Michele H Duarte, Miguel T De Lanna, Milton S C Neto, Mirelly C Quites, Mirtes M M R Bertozzi, Moises Carminatti, Natalia B Schuch, Natalia C V De Melo, Nayanne M Homem, Neide M Murai, Nicola O Oliva, Pablo M Chaves, Patricia D S Pinto, Patricia V Lima, Paula V Salgado, Pauline N Lobao, Paulo Caldas, Paulo G D A Suassuna, Rafael F Romani, Rafael Pavani, Rafaela Zampier, Raiza R D C Rocha, Ramon R Filho, Raphael G Andrade, Raquel G Siqueira, Raysa S A Barbosa, Renata D C Zen, Reuber S Junior, Ricardo A Mothe, Ricardo Benevenutti, Roberto E Salum, Roberto K G De Oliveira, Roberto S S Santos, Rodrigo A Wanderley, Rodrigo Braz, Rodrigo C Rios, Rodrigo G Da Silva, Rodrigo O Nobre, Rodrigo R Mion, Rodrigo V M De Paula, Rogeria A S Fernandes, Rolando G V Valenzuela, Ronaldo G Da Silva, Rubia B B Boaretto, Sabrina B Polycarpo, Sabrina C D E Martins, Sandra S Vilaca, Sergio M D S Junior, Sergio R R De Araujo, Sidnei C Brandao, Silvia C F Medeiros, Silvia D V Figueiredo, Silvia M Botelho, Simone C Lo, Stanley D A Araujo, Stenio B De Freitas, Sylvia A D Turani, Tania M S Fontes, Tatiana V Carneiro, Teodoro Ostrowski, Tgustavo N Betonico, Thais P Torres, Thatyana Wendhausen, Theo R Costa, Thiago F Nunes, Thiago L Ataides, Thiago Reis, Thomaz Antunes, Tiago L Cerqueira, Tomas P Junior, Tulio C Carvalho, Tulio H E Salem, Ursula D S Teixeira, Ursula S Mendonca, Valencio P De Carvalho, Vanesa C B Silva, Vanessa C D B Silva, Vanessa D S Silva, Vanessa G B Rodrigues, Victor E P Pinto, Vinicius S Colares, Vitor E S Sousa, Vivane A Leite, Vivian S Teixeira, Viviane C Ponciano, Walid K Omais, Wallace Pereira, Washington L C D Santos, Wendell F De Moura, Willians V D Rodrigues, Winnie M C Beilner.
