# Supplementary material for: The Brazilian registry of kidney biopsies: results from the 2022 national prospective registry
Source: J Bras Nefrol. 2026 Oct 2;48(4):e20260096. doi: 10.1590/2175-8239-JBN-2026-0096en (PMC13645486; doi:10.1590/2175-8239-JBN-2026-0096en)
Supplement: Translation

# Translation: Registro brasileiro de biópsias renais: resultados do registro nacional prospectivo de 2022

**DOI:** 10.1590/2175-8239-JBN-2026-0096pt

**Authors:** Irene L. Noronha, Rodrigo José Ramalho, Claudia Maria Costa de Oliveira, Marilia Bahiense-Oliveira, Fabricio Augusto Marques Barbosa, Jose de Resende Barros, Ronny Mitsuoka, Osvaldo Merege Vieira-Neto, Precil Diego Miranda de Menezes Neves

**Affiliations:** 1 Sociedade Brasileira de Nefrologia, Departamento de Nefrologia Clínica, São Paulo, SP, Brasil.; 2 Universidade de São Paulo, Hospital das Clínicas, Faculdade de Medicina, Divisão de Nefrologia, São Paulo, SP, Brasil.; 3 Faculdade de Medicina de São José do Rio Preto, Divisão de Nefrologia, São Paulo, SP, Brasil.; 4 Hospital Universitário Walter Cantídio, Divisão de Nefrologia, Fortaleza, CE, Brasil.; 5 Universidade de São Paulo, Faculdade de Medicina de São Paulo, Hospital das Clínicas, Instituto de Radiologia, São Paulo, SP, Brasil.; 6 Universidade de São Paulo, Faculdade de Medicina de Ribeirão Preto, Departamento de Clínica Médica, Ribeirão Preto, SP, Brasil.

**Keywords:** Biópsia Renal, Doenças Glomerulares, Patologia Renal, Registro, Brasil

## Abstract

**Introdução:**

A biópsia renal é fundamental para o diagnóstico etiológico e o manejo das doenças renais. O Registro Brasileiro de Biópsias Renais (RBBR), criado pela Sociedade Brasileira de Nefrologia, visa caracterizar o perfil clínico e histopatológico das biópsias renais realizadas no Brasil. O objetivo deste artigo é apresentar os dados do RBBR de 2022.

**Métodos:**

Análise dos dados de pacientes adultos submetidos à biópsia de rim nativo e incluídos prospectivamente no RBBR entre janeiro e dezembro de 2022. Foram avaliados dados demográficos, clínicos, laboratoriais e histopatológicos.

**Resultados:**

Foram incluídas 1.142 biópsias renais provenientes de centros de todas as regiões do país. Houve discreto predomínio do sexo feminino (54,8%), com idade média de 44,8 ± 16,4 anos. As principais indicações para biópsia foram disfunção renal (51,8%) e síndrome nefrótica (39,4%). Hipertensão arterial e diabetes mellitus estavam presentes em 47,8% e 15,1% dos pacientes, respectivamente. No momento da biópsia, 67,1% apresentavam taxa de filtração glomerular estimada < 60 mL/min/1,73 m^2^. As doenças glomerulares corresponderam a 72,5% dos diagnósticos histológicos, sendo a nefrite lúpica (23,1%), a nefropatia por IgA (15,0%) e a glomeruloesclerose segmentar e focal (11,2%) as mais frequentes.

**Conclusão:**

Os dados de 2022 do RBBR confirmam a predominância das doenças glomerulares entre as biópsias renais no Brasil e reforçam a relevância do registro como ferramenta para a compreensão da epidemiologia das doenças renais no país. Entre os pacientes diabéticos submetidos à biópsia, apenas metade apresentou evidências histológicas de nefropatia diabética, o que ressalta a importância da biópsia renal em casos selecionados.

## INTRODUÇÃO

A doença renal crônica (DRC) representa um importante desafio global de saúde pública, acometendo aproximadamente 10–15% da população adulta mundial e apresentando aumento sustentado de sua prevalência nas últimas décadas^1,2^. A hipertensão arterial e o diabetes mellitus (DM) permanecem como as principais causas de DRC globalmente; entretanto, as doenças glomerulares continuam representando entre a segunda e a terceira causas mais frequentes de doença renal com necessidade de terapia renal substitutiva (DRC-TRS) em muitas regiões^3,4^.

Além de sua contribuição para a DRC-TRS, as doenças glomerulares estão associadas à morbidade significativa, a trajetórias clínicas heterogêneas e a respostas variáveis ao tratamento. Nos últimos anos, avanços na imunopatologia, no diagnóstico molecular e em terapias direcionadas redesenharam o campo das doenças glomerulares, reforçando a importância do diagnóstico etiológico preciso e de uma caracterização epidemiológica refinada^5,6^. Nesse contexto, a biópsia renal permanece como ferramenta fundamental para o diagnóstico, a estratificação prognóstica e a tomada de decisões terapêuticas em pacientes com suspeita de doença glomerular.

No Brasil, inquéritos nacionais sobre diálise revelam consistentemente que as doenças glomerulares representam aproximadamente 5% a 9% dos pacientes em terapia de substituição renal, figurando entre as principais causas de DRC avançada não diabética no país^4^. Apesar dessa relevância, dados nacionais abrangentes sobre doenças renais comprovadas por biópsia são historicamente escassos. A maioria das evidências disponíveis provém de experiências de centros únicos ou de coortes regionais, limitando a capacidade de descrever padrões em nível nacional, avaliar a variabilidade regional ou orientar o planejamento de políticas de saúde^7,8,9,10,11^.

Estudos brasileiros prévios, incluindo grandes registros regionais e séries institucionais, forneceram informações importantes sobre a epidemiologia local das doenças glomerulares, mas foram limitados pelo escopo geográfico e pela heterogeneidade temporal^7,8,9,10,11^. Além disso, mudanças nas práticas diagnósticas, incluindo o uso mais amplo da imunofluorescência, da microscopia eletrônica e de biomarcadores específicos para cada doença, provavelmente modificaram o espectro das doenças glomerulares observado ao longo do tempo, ressaltando a necessidade de dados contemporâneos e coletados de forma sistemática.

No intuito de suprir essa lacuna, a Sociedade Brasileira de Nefrologia (SBN), por meio do Departamento de Nefrologia Clínica, criou o Registro Brasileiro de Biópsias Renais (RBBR)^12^, um registro nacional, multicêntrico e prospectivo, destinado a coletar dados clínicos e histopatológicos padronizados de biópsias de rins nativos realizadas em todo o país. O registro tem como objetivo fornecer uma base epidemiológica robusta para a caracterização das doenças renais no Brasil, com ênfase especial nas doenças glomerulares.

A primeira publicação do RBBR descreveu os dados do primeiro ano de implementação do registro, incluindo 954 biópsias realizadas em 2021, e demonstrou a viabilidade da coleta de dados em âmbito nacional^12^. Com base nesse relato, o presente estudo apresenta os resultados do segundo ano do registro, abrangendo as biópsias renais realizadas em 2022. Ao ampliar o conjunto de dados e comparar os resultados entre anos consecutivos, esta análise busca fornecer uma visão geral mais abrangente e atualizada da epidemiologia das doenças renais comprovadas por biópsia no Brasil e consolidar ainda mais o RBBR como uma plataforma de referência para pesquisa clínica e para o desenvolvimento de políticas de saúde no país.

## MÉTODOS

### Desenho do estudo e estrutura do registro

O RBBR é um registro nacional, multicêntrico, prospectivo e observacional, coordenado pela SBN por meio do Departamento de Nefrologia Clínica. Tal registro foi idealizado para coletar sistematicamente dados clínicos e histopatológicos padronizados de biópsias de rim nativo realizadas em pacientes adultos em todo o Brasil.

Os dados são inseridos em uma plataforma eletrônica segura, hospedada pela SBN (https://www.glomerulopatias-sbn.org.br/inicio). O sistema é suportado por um banco de dados MySQL com protocolos de segurança criptografados (SSL) e não armazena informações que permitam a identificação direta dos pacientes. Para minimizar registros duplicados, a plataforma realiza uma verificação cruzada automatizada com base nas iniciais do paciente, na data de nascimento e no sexo. Os centros participantes têm acesso apenas aos seus próprios dados, enquanto o gerenciamento centralizado dos dados, a extração e a geração de relatórios são conduzidos por um profissional de ciência de dados afiliado à SBN.

Os nefrologistas participantes foram recrutados por meio do Departamento de Nefrologia Clínica da Sociedade Brasileira de Nefrologia (SBN), que envolveu representantes das filiais regionais da SBN e nefrologistas com expertise em doenças glomerulares de todas as cinco regiões geográficas do Brasil. A participação foi voluntária e promovida por meio de comunicação direta, apresentações em congressos nacionais de nefrologia e canais de comunicação da SBN. Uma vez que as biópsias renais no Brasil são frequentemente analisadas por laboratórios de nefropatologia de referência que atendem a diversos hospitais e consultórios de nefrologia, o número exato de centros participantes não pôde ser determinado com precisão utilizando uma estrutura convencional baseada em centros. Durante a fase inicial de implementação, 45 centros universitários de nefrologia, juntamente com outros serviços privados e não universitários, contribuíram com casos de biópsia para o registro.

Este manuscrito foi elaborado de acordo com as diretrizes *Strengthening the Reporting of Observational Studies in Epidemiology* (STROBE) para estudos observacionais.

### População do estudo e critérios de inclusão

A presente análise inclui dados de pacientes adultos (≥18 anos) que foram submetidos à biópsia de rim nativo entre 1º de janeiro e 31 de dezembro de 2022 e cujas informações foram registradas prospectivamente no RBBR. Não foram incluídas biópsias pediátricas nem biópsias de transplante renal. Quando mais de uma biópsia foi realizada no mesmo paciente, apenas a primeira biópsia foi considerada para análise. As biópsias renais foram realizadas de acordo com as indicações clínicas estabelecidas pelos nefrologistas responsáveis em cada centro participante, refletindo a prática clínica de rotina.

### Coleta de dados e variáveis do estudo

Os dados clínicos e demográficos foram coletados no momento da realização da biópsia renal por meio de um questionário eletrônico padronizado. As variáveis registradas incluíram idade, sexo, raça autodeclarada, data da biópsia, estado em que o procedimento foi realizado e região de origem. A raça foi registrada utilizando-se categorias predefinidas disponíveis no sistema de captura eletrônica de dados (branca, negra, parda, asiática ou indígena).

As indicações clínicas para a realização da biópsia renal foram categorizadas como síndrome nefrótica, proteinúria não nefrótica, hematúria, glomerulonefrite rapidamente progressiva ou disfunção renal. A disfunção renal foi registrada quando o nefrologista responsável pelo tratamento considerou a função renal comprometida como uma das indicações para a biópsia renal. O registro não fez distinção entre lesão renal aguda, progressão da DRC ou outras causas de comprometimento da função renal. Foram coletadas também informações sobre comorbidades, incluindo hipertensão e DM previamente diagnosticadas, bem como histórico pessoal ou familiar de doença renal. Tais variáveis foram registradas como presentes, ausentes ou desconhecidas. Não foram estabelecidos limites laboratoriais para essas indicações, refletindo a avaliação clínica do nefrologista responsável pelo tratamento.

Os valores de creatinina sérica no momento da biópsia foram registrados, e a taxa de filtração glomerular estimada (TFGe) foi calculada automaticamente pela plataforma.

### Avaliação histopatológica

Os diagnósticos histopatológicos foram estabelecidos por nefropatologistas locais com base na microscopia óptica e na imunofluorescência, sendo realizada a microscopia eletrônica quando disponível. O número de glomérulos presentes em cada amostra de biópsia foi registrado. A fibrose intersticial e a atrofia tubular foram avaliadas de forma semiquantitativa e categorizadas como ausente, leve (< 25%), moderada (25–50%) ou grave (> 50%).

As análises de imunofluorescência e/ou imuno-histoquímica incluíram a avaliação de IgA, IgG, IgM, C3, C1q, cadeias leves kappa (κ) e lambda (λ), fibrinogênio e anticorpos anti-PLA2R, com documentação da localização e intensidade dos depósitos.

Mais de um diagnóstico histológico poderia ser registra­do para uma determinada biópsia. Nesses casos, um diagnóstico histológico primário era definido com base na revisão pelo comitê coordenador, em consulta com o nefrologista e/ou nefropatologista responsável pelo encaminhamento, e esse diagnóstico foi utilizado para as análises epidemiológicas.

### Classificação dos diagnósticos histológicos

Para fins analíticos, os diagnósticos histológicos foram inicialmente agrupados em oito categorias principais: doenças glomerulares, doenças vasculares, doenças tubulointersticiais, gamopatias monoclonais, nefropatia diabética, doenças renais hereditárias, diagnósticos diversos e biópsias não classificáveis.

Opções diagnósticas detalhadas foram disponibilizadas na plataforma eletrônica para garantir a consistência do preenchimento dos dados entre os centros. Os diagnósticos foram selecionados a partir de listas predefinidas elaboradas pelos membros do Departamento de Nefrologia Clínica da SBN.

Essas categorias diagnósticas amplas foram adotadas para facilitar a geração de relatórios epidemiológicos e a comparação das frequências das doenças em todo o registro. Reconhece-se que certas doenças podem ser classificadas em mais de uma categoria patológica; portanto, a classificação foi concebida como uma estrutura pragmática para análises do registro, e não como uma taxonomia estritamente fisiopatológica.

### Análise estatística

As variáveis contínuas foram descritas como média ± desvio-padrão ou mediana com intervalo interquartil, de acordo com a distribuição dos dados. Já as variáveis categóricas foram expressas como frequências absolutas e porcentagens. As análises estatísticas foram realizadas utilizando o software SPSS (versão 29.0, IBM Corp.).

### Considerações éticas

O protocolo do estudo RBBR foi aprovado pelo Comitê de Ética da Faculdade de Medicina de São José do Rio Preto (CAAE: 59947022.6.0000.5415; aprovação nº 5.586.556). Trata-se de um registro aprovado como uma iniciativa multicêntrica nacional coordenada pela Sociedade Brasileira de Nefrologia. O RBBR é um registro observacional e prospectivo, e o presente estudo consistiu em uma análise retrospectiva de dados anonimizados previamente coletados no âmbito do registro. Como as análises foram realizadas utilizando dados cadastrais desidentificados, sem contato direto com os pacientes ou qualquer intervenção, a exigência de consentimento informado foi dispensada pelo Comitê de Ética.

## RESULTADOS

### Representatividade nacional da coorte

Em 2022, um total de 1.142 biópsias de rim nativo foram incluídas no RBBR, provenientes de centros localizados em todas as cinco regiões geográficas do Brasil. Os estados com o maior número de biópsias registradas foram São Paulo, Minas Gerais e Bahia. A maioria dos pacientes era da região Sudeste (63,9%), seguida pela região Nordeste (24,5%). A distribuição das biópsias por estado (números absolutos) e por região geográfica (proporções) é apresentada na Figura 1.

**Figura 1:** Distribuição geográfica das biópsias renais incluídas no Registro Brasileiro de Biópsias Renais (RBBR) em 2022. (A) Número total de biópsias renais por estado onde o procedimento foi realizado. (B) Proporção de biópsias nas cinco regiões geográficas do Brasil.

### Características demográficas

A idade média dos pacientes à biópsia foi de 44,8 ± 16,4 anos, com a maior concentração na faixa etária de 31 a 40 anos (23%) (Figura 2). Houve uma discreta predominância de pacientes do sexo feminino (54,8%). Em relação à raça autodeclarada, 61,7% dos pacientes se identificaram como pardos e 32,5% como brancos; os pacientes negros representaram 5,0% e os asiáticos, 0,8%. Não foram registrados pacientes indígenas em 2022.

**Figura 2:** Distribuição das biópsias renais por faixa etária no Registro Brasileiro de Biópsias Renais (RBBR) em 2022.

### Características clínicas e laboratoriais

A indicação mais comum para a biópsia renal foi disfunção renal (51,8%), seguida de síndrome nefrótica (39,4%) (Figura 3). No momento da biópsia, 47,8% dos pacientes apresentavam hipertensão e 15,1% eram diabéticos. História familiar de doença renal foi relatada em 4,7% dos casos. A função renal estava frequentemente comprometida à biópsia: 67,1% dos pacientes apresentavam TFGe < 60 mL/min/1,73 m^2^, e 15,0% < 15 mL/min/1,73 m^2^ (Figura 4).

**Figura 3:** Distribuição do quadro clínico à biópsia renal no Registro Brasileiro de Biópsias Renais (RBBR) em 2022.

**Figura 4:** Distribuição dos pacientes de acordo com a taxa de filtração glomerular estimada (TFGe) e o estágio da DRC à biópsia renal no Registro Brasileiro de Biópsias Renais (RBBR) em 2022. Os valores acima das barras indicam a porcentagem do total de biópsias incluídas no registro.

### Características gerais das amostras de biópsia renal

A maioria das biópsias renais apresentou representativi­dade histológica adequada, com 78,5% contendo ≥ 10 glomérulos avaliáveis (Figura 5A). A fibrose intersticial e a atrofia tubular leves foram observadas em 60,2% das biópsias (Figura 5B). Todos os casos foram submetidos à avaliação por imunofluorescência e/ou imuno-histoquímica. A microscopia eletrônica foi realizada em apenas 6% dos pacientes.

**Figura 5:** Características histopatológicas das biópsias renais incluídas no Registro Brasileiro de Biópsias Renais (RBBR) em 2022. (A) Número de glomérulos identificados por amostra de biópsia. (B) Grau de fibrose intersticial e atrofia tubular (FIAT), categorizado como leve (grau 1), moderado (grau 2) ou grave (grau 3).

### Diagnósticos histológicos

As doenças glomerulares foram a categoria diagnóstica mais frequente, representando 72,5% de todas as biópsias renais. As categorias mais comuns na sequência foram a doença renal diabética (6,4%) e as doenças tubulointersticiais (3,5%). Em 11% dos casos, não foi possível estabelecer um diagnóstico histológico conclusivo (Figura 6). A maioria das biópsias reportadas como “inclassificáveis” não se devia a amostras inadequadas. Em vez disso, correspondiam predominantemente à DRC avançada, caracterizada por glomeruloesclerose global extensa, atrofia tubular, fibrose intersticial e dano parenquimatoso crônico irreversível, o que impedia um diagnóstico etiológico específico. Uma proporção menor consistia em nefrite tubulointersticial crônica com características inespecíficas, lesões renais crônicas inespecíficas ou amostras de biópsia subótimas.

**Figura 6:** Distribuição dos diagnósticos histológicos de acordo com as principais categorias de doenças no Registro Brasileiro de Biópsias Renais (RBBR) em 2022. Para fins analíticos, os diagnósticos foram agrupados em oito categorias diagnósticas principais. Os valores indicam a porcentagem de todas as biópsias incluídas no registro.

Entre as doenças glomerulares, a nefrite lúpica (NL) foi o diagnóstico mais frequente, representando 23,1% dos casos, seguida de nefropatia por IgA (NIgA) (15,0%), glomeruloesclerose segmentar e focal (GESF) (11,2%) e nefropatia membranosa (NM) (9,5%) (Figura 7; Tabela 1).

**Figura 7:** Espectro e frequência das doenças glomerulares comprovadas por biópsia no Registro Brasileiro de Biópsias Renais (RBBR) em 2022. A figura mostra a distribuição dos diagnósticos histológicos entre as biópsias classificadas como doenças glomerulares. As porcentagens são expressas em relação ao número total de biópsias incluídas no registro.

**Tabela 1:** Distribuição dos diagnósticos histológicos no Registro Brasileiro de Biópsias Renais (RBBR) em 2022, agrupados de acordo com as principais categorias diagnósticas.

Doenças glomerulares	n = 828	72,5%
Nefrite lúpica	264	23,1%
Nefropatia por IgA	171	15,0%
Glomeruloesclerose segmentar e focal	128	11,2%
Nefropatia membranosa	108	9,5%
Vasculite pauci-imune	45	3,9%
GN membranoproliferativa mediada por imunocomplexos	20	1,8%
Doença de lesões mínimas	18	1,6%
Glomerulopatia colapsante	18	1,6%
Glomerulopatia do C3	15	1,3%
Microangiopatia trombótica	13	1,1%
GN crescêntica mediada por imunocomplexos	9	0,8%
GN proliferativa mesangial	8	0,7%
GN aguda pós-infecciosa	4	0,4%
GN crioglobulinêmica	3	0,3%
GN por anticorpo anti-membrana basal glomerular	2	0,2%
GN relacionada à infecção	2	0,2%
**Nefropatia diabética**	**n = 73**	**6,4%**
**Doenças tubulointersticiais**	**n = 40**	**3,5%**
Nefrite intersticial aguda	25	2,2%
Necrose tubular aguda	15	1,6%
**Gamopatias monoclonais**	**n = 36**	**3,2%**
Amiloidose	17	1,5%
Nefropatia por cilindros de cadeia leve	6	0,6%
Doença por depósito de imunoglobulina monoclonal	4	0,4%
Tubulopatia proximal por cadeia leve	1	0,1%
Outras gamopatias monoclonais	3	0,3%
GN proliferativa com depósitos de imunoglobulina monoclonal	3	0,3%
Glomerulonefrite fibrilar	2	0,2%
**Doenças vasculares**	**n = 24**	**2,1%**
Nefroesclerose hipertensiva	23	2,0%
Doença renal associada à esclerose sistêmica	1	0,1%
Doenças renais hereditárias	2	0,2%
Doenças relacionadas ao colágeno tipo IV	2	0,2%
**Miscelânia**	**n = 13**	**1,1%**
**Inclassificáveis**	**n = 126**	**11%**

Abreviatura – GN: glomerulonefrite.

A estratificação por idade demonstrou maiores frequências de NL e GESF entre indivíduos mais jovens. Na faixa etária de 31 a 50 anos, a NL e a NIgA foram os diagnósticos predominantes, enquanto a NM e a nefropatia diabética (ND) foram observadas com maior frequência em faixas etárias mais avançadas (Figura 8).

**Figura 8:** Distribuição estratificada por idade das doenças glomerulares mais frequentes comprovadas por biópsia no Registro Brasileiro de Biópsias Renais (RBBR). A figura ilustra a distribuição da nefrite lúpica, GESF/DLM, NIgA, nefropatia membranosa e nefropatia diabética em três faixas etárias (18–30 anos, 31–50 anos e ≥ 51 anos). Os valores acima das barras indicam a porcentagem de todas as biópsias incluídas no registro.

No que diz respeito à distribuição dos diagnósticos por sexo, a NL, como esperado, apresentou uma predominância acentuada entre as pacientes do sexo feminino, enquanto a maioria das outras doenças glomerulares foi ligeiramente mais prevalente entre os homens (Figura 9).

**Figura 9:** Distribuição por sexo das doenças glomerulares mais frequentes comprovadas por biópsia no Registro Brasileiro de Biópsias Renais (RBBR). Os valores acima das barras indicam a porcentagem de pacientes do sexo masculino e feminino em cada categoria de diagnóstico. O número total de casos para cada diagnóstico é apresentado abaixo.

A Figura 10 ilustra o quadro clínico apresentado no momento da biópsia renal em relação às glomerulopatias mais comuns. A síndrome nefrótica foi a apresentação mais frequente entre pacientes com glomeruloesclerose segmentar e focal/doença de lesões mínimas (GESF/DLM) e NM, sendo também comumente observada em pacientes com ND. Em contrapartida, a proteinúria não nefrótica e a hematúria foram mais frequentes entre pacientes com NL e NIgA. Notavelmente, com exceção dos pacientes com GESF/DLM, a maioria dos pacientes nos demais grupos já apresentava disfunção renal à biópsia renal.

**Figura 10:** Frequência das apresentações clínicas caracterizadas por síndrome nefrótica, proteinúria não nefrótica, hematúria ou disfunção renal entre pacientes com as doenças glomerulares mais comuns comprovadas por biópsia. Os valores acima das barras indicam a porcentagem de pacientes em cada categoria de diagnóstico histológico.

### Pacientes diabéticos submetidos à biópsia renal

Na presente coorte, 164 pacientes (14,4%) apresentavam diagnóstico de DM no momento da biópsia renal (Figura 11). Notavelmente, entre esses, apenas 82 (50,0%) apresentaram características histológicas de ND, enquanto os 82 pacientes restantes (50,0%) não apresentaram evidência histológica da doença.

**Figura 11:** Achados histológicos em pacientes com diabetes mellitus submetidos à biópsia renal no Registro Brasileiro de Biópsias Renais (RBBR) em 2022. Entre 164 pacientes diabéticos, 82 (50%) apresentaram características histológicas de nefropatia diabética, enquanto os demais 82 (50%) não apresentaram evidência histológica da doença. Em 73 casos, a nefropatia diabética foi o principal diagnóstico histológico, enquanto, em 9, foi identificada como diagnóstico histológico associado.

Entre os pacientes com ND, tal condição foi considerada o diagnóstico histológico primário em 73 casos (89%), correspondendo a 6,4% de todas as biópsias renais incluídas no registro. Em quatro desses pacientes (5,5%), foram identificados achados histológicos adicionais, incluindo nefrite intersticial aguda (n = 2), nefrosclerose hipertensiva (n = 1) e podocitopatia (n = 1). Nos 9 casos restantes, a ND foi identificada como um achado histológico associado a outra doença renal primária, incluindo NIgA (n = 4), glomerulopatia colapsante (n = 2), GESF (n = 1), NM (n = 1) e glomerulonefrite membranoproliferativa mediada por complexos imunes (n = 1).

A idade média à biópsia foi semelhante entre os pacientes diabéticos com e sem ND (57,9 ± 11,6 vs. 56,8 ± 12,2 anos, respectivamente), assim como a distribuição por sexo. Os pacientes com ND apresentavam, com maior frequência, insuficiência renal no momento da biópsia (67,1% vs. 39,9%). A síndrome nefrótica foi a apresentação clínica mais comum entre tais pacientes (46,3%), enquanto a hematúria foi observada com maior frequência entre os pacientes diabéticos sem ND (19,5% vs. 12,2%).

Os pacientes com ND também apresentaram compro­metimento mais grave da função renal no momento da biópsia. A mediana da creatinina sérica foi significativamente maior nos pacientes com ND em comparação àqueles sem a doença (2,56 [IQR 1,72–4,11] vs. 1,63 [IQR 1,17–2,43] mg/dL; *p* = 0,0001), enquanto a mediana da TFGe foi significativamente menor (24,9 [IQR 15,3–40,2] vs. 41,5 [IQR 24,7–62,7] mL/min/1,73 m^2^; *p* = 0,0003).

É importante destacar que, em comparação com 2021, não houve relato de pacientes com nefropatias associadas ao HIV, doença renal relacionada à IgG4, glomerulonefrite imunotactoide, doença de Fabry ou deficiência de LCAT. A maioria são doenças raras e, para muitas delas, a microscopia eletrônica é essencial para o diagnóstico. Nesse contexto, o acesso limitado à microscopia eletrônica pode representar uma limitação significativa para o estabelecimento desses diagnósticos.

## DISCUSSÃO

Dados epidemiológicos confiáveis sobre doenças glomerulares continuam limitados em muitos países de renda média, apesar do fardo substancial da DRC e da ampla disponibilidade de terapias de substituição renal^2,13^. No Brasil, grandes registros nacionais têm caracterizado com sucesso a atividade de diálise e transplante^4,14^; no entanto, as informações derivadas de biópsias renais em pacientes com rins nativos têm sido, historicamente, fragmentadas e amplamente restritas a séries de centro único ou a estudos regionais^7,15,16,17^. Nesse contexto, o RBBR representa um passo fundamental para a coleta sistemática de dados baseados em biópsias em nível nacional.

A presente análise, correspondente ao segundo ano do RBBR, reflete a transição do registro de uma fase inicial de implementação para uma plataforma de coleta de dados consolidada^12^. Em 2022, observou-se um aumento substancial no número de biópsias renais registradas em comparação com o ano inaugural, o que ressalta o maior envolvimento dos centros participantes e a melhor integração do registro à prática clínica de rotina. Fases de maturação semelhantes já foram descritas em outros registros nacionais e regionais de biópsias. É importante ressaltar que essa expansão ocorreu sem grandes mudanças na distribuição geográfica dos casos, mantendo-se a predominância da região Sudeste, seguida pela região Nordeste. Em vez de indicar apenas um viés de amostragem, esse padrão provavelmente reflete a distribuição desigual dos serviços de nefrologia e dos centros de referência em todo o país.

Além do seu crescimento quantitativo, o conjunto de dados do RBBR de 2022 oferece uma estrutura mais robusta para descrever o panorama atual das indicações e dos diagnósticos de biópsia renal no Brasil. A estabilidade das características demográficas e da representação regional, somada ao aumento do tamanho da amostra, fortalece a consistência interna do registro e sustenta seu uso como fonte de referência para futuras investigações epidemiológicas e clínicas. À medida que o RBBR continua a se expandir, seu potencial longitudinal poderá permitir análises mais refinadas de tendências temporais, variabilidade regional e desfechos específicos das doenças, aumentando ainda mais sua relevância para a tomada de decisões clínicas e o planejamento de políticas de saúde.

A distribuição etária dos pacientes submetidos à biópsia renal em 2022 refletiu fielmente a observada durante o primeiro ano do registro, com predominância de adultos jovens e de meia-idade. Perfis etários semelhantes foram relatados em registros de biópsias da América Latina e do Sul da Europa^18^. A ligeira predominância de pacientes do sexo feminino difere dos padrões relatados em vários registros internacionais de biópsias^19,20^, mas é plausivelmente explicada pela alta frequência sustentada de NL na coorte brasileira. Ao comparar o conjunto de dados de 2022 com o relatório do BRKB de 2021, surgem várias observações relevantes. Em primeiro lugar, houve um aumento substancial no número de biópsias registradas, refletindo a consolidação progressiva do registro e uma participação mais ampla dos centros. Apesar dessa expansão, as características demográficas, as indicações clínicas e a distribuição geral das principais categorias diagnósticas permaneceram notavelmente estáveis, corroborando a consistência interna da plataforma.

A raça autodeclarada no Brasil reflete uma população altamente miscigenada, moldada por diversas contribuições ancestrais^21,22^. Na presente coorte, uma alta proporção de pacientes se autodeclarou como parda, superando a proporção relatada no primeiro ano do registro. Em vez de sugerir um agrupamento racial específico para a doença, esse achado provavelmente reflete uma participação geográfica mais ampla e uma maior completude dos dados demográficos registrados em 2022. Tais observações reforçam a utilidade limitada da raça autodeclarada como indicador substituto da ascendência genética em estudos epidemiológicos de doença renal no Brasil^22,23^. Notavelmente, a proporção de biópsias reportadas como “inclassificáveis” aumentou em 2022. Uma análise detalhada desses casos demonstrou que a maioria não resultou de amostras de biópsia inadequadas ou de divergência diagnóstica entre os nefropatologistas. Em vez disso, a maioria correspondia à DRC avançada, com glomeruloesclerose global extensa, atrofia tubular, fibrose intersticial e dano parenquimatoso crônico irreversível, o que impediu a identificação de uma etiologia subjacente específica. Uma proporção menor consistiu em nefrite tubulointersticial crônica com características inespecíficas ou lesões consideradas insuficientemente características para sustentar um diagnóstico definitivo. Entretanto, o acesso limitado à microscopia eletrônica também pode ter contribuído para a incerteza diagnóstica em um subconjunto de casos, particularmente em doenças nas quais a avaliação ultraestrutural é importante para estabelecer um diagnóstico definitivo ou refinar a classificação histopatológica.

No que diz respeito às indicações clínicas, a disfunção renal continuou sendo o principal motivo para a realização de uma biópsia de rim nativo, seguida pela síndrome nefrótica, reproduzindo o padrão geral observado em 2021 e em outros grandes registros de biópsias^24,25^. Notavelmente, mais de dois terços dos pacientes apresentavam uma TFGe inferior a 60 mL/min/1,73 m^2^ no momento da biópsia, indicando que uma proporção substancial dos procedimentos foi realizada em indivíduos com DRC já estabelecida. Achados semelhantes foram relatados em coortes contemporâneas, refletindo uma tendência à realização tardia de biópsias na prática clínica de rotina^26^. Apesar disso, a predominância de fibrose intersticial e atrofia tubular leves sugere que muitos pacientes foram submetidos à biópsia em estágios nos quais o esclarecimento diagnóstico ainda poderia influenciar o manejo e, potencialmente, modificar a trajetória da doença. Tais achados ressaltam a relevância contínua da biópsia renal em pacientes com redução da função renal, particularmente quando o quadro clínico é atípico ou persiste a incerteza diagnóstica^27,28^.

Em termos de espectro da doença, a NL continuou sendo o diagnóstico mais frequente em ambos os anos do RBBR. Observou-se um aumento modesto na frequência relativa da NIgA em 2022, reforçando ainda mais sua relevância epidemiológica no Brasil e possivelmente refletindo um melhor reconhecimento da doença à medida que a cobertura do registro se expande. É importante ressaltar que as frequências relativas de outros diagnósticos glomerulares importantes, incluindo a GESF e a NM, permaneceram amplamente estáveis, sugerindo a preservação do perfil diagnóstico geral, apesar da expansão do registro.

No segundo ano do RBBR, as doenças glomerulares continuaram responsáveis pela maioria dos diagnósticos histológicos, representando quase três quartos de todas as biópsias realizadas. Esse padrão é consistente com o observado na maioria dos registros nacionais e internacionais de biópsias^3,4,29^. A persistência dessa distribuição reforça o papel central das doenças glomerulares como principal indicação para a biópsia de rim nativo no Brasil. Ao mesmo tempo, a maior proporção de biópsias classificadas como inconclusivas destaca os desafios diagnósticos persistentes, que podem estar relacionados a diferenças na complexidade dos casos, à disponibilidade de técnicas auxiliares ou à variabilidade dos fluxos diagnósticos entre os centros participantes^24^.

No âmbito das doenças glomerulares, a NL continuou sendo o diagnóstico mais frequente, seguida pela NIgA e pela GESF. Essa distribuição se assemelha à relatada em diversas coortes latino-americanas, mas difere dos padrões observados no Leste Asiático e em partes da Europa, onde a NIgA predomina^30,31^. O aumento da incidência de NIgA em 2022 reforça ainda mais sua relevância como uma das principais causas de doença glomerular no Brasil, em consonância com o reconhecimento mais amplo dessa entidade à medida que as capacidades diagnósticas melhoram e a cobertura dos registros se amplia^6^.

Os padrões relacionados ao sexo e à idade observados em 2022 estavam em consonância com o comportamento biológico das doenças subjacentes. A NL predominou entre as mulheres, enquanto outras doenças glomerulares foram diagnosticadas com maior frequência nos homens. As análises estratificadas por idade revelaram padrões distintos entre as principais doenças glomerulares. A NL predominou entre adultos mais jovens, particularmente entre 18 e 50 anos, refletindo a epidemiologia conhecida do lúpus eritematoso sistêmico. Em contrapartida, a NM e a ND foram substancialmente mais frequentes entre indivíduos mais velhos, particularmente naqueles com mais de 50 anos. A NIgA apresentou um pico de frequência em adultos de meia-idade, enquanto a GESF e a DLM se distribuíram por todas as faixas etárias. Esses achados são amplamente consistentes com registros internacionais de biópsias e corroboram a plausibilidade biológica do conjunto de dados do RBBR^18,32,33,34^.

Embora a doença renal diabética tenha representado uma minoria dos diagnósticos histológicos, sua contribuição continua sendo clinicamente significativa. Notavelmente, quase metade dos pacientes diabéticos submetidos à biópsia renal não apresentou características histológicas compatíveis com ND, e uma proporção substancial apresentou diagnósticos sobrepostos ou alternativos, sendo a NIgA o mais comum. Observações semelhantes têm sido consistentemente relatadas em estudos baseados em biópsias de pacientes diabéticos^35,36,37^. Tais achados ressaltam a importância contínua da biópsia renal em pacientes selecionados com diabetes, particularmente quando o quadro clínico é atípico ou o curso da doença se desvia do padrão esperado^38^.

É importante ressaltar que os pacientes diabéticos submetidos à biópsia renal não representam a população geral de indivíduos com DM. Na prática clínica, a biópsia renal é geralmente reservada para pacientes com quadros clínicos atípicos, deterioração rápida da função renal, sedimento urinário ativo, síndrome nefrótica ou suspeita de doença renal sobreposta. Portanto, a alta proporção de doenças renais não diabéticas e de padrões histológicos mistos observada na presente coorte deve ser interpretada no contexto de uma população selecionada para biópsia, e não como representativa da população de pacientes diabéticos em geral. No entanto, esses achados reforçam o valor diagnóstico da biópsia renal em pacientes diabéticos selecionados e destacam as limitações da utilização exclusiva de critérios clínicos para o diagnóstico da doença renal diabética.

Uma consideração adicional diz respeito à organização dos serviços de biópsia renal no Brasil. Ao contrário dos registros de diálise, as biópsias renais são frequentemente processadas em laboratórios de nefropatologia de referência que recebem amostras de diversos hospitais e consultórios de nefrologia. Consequentemente, o número de centros participantes não pode ser definido com precisão por meio de uma abordagem convencional baseada em centros. Embora a participação tenha sido voluntária e as contribuições regionais tenham sido desiguais, os casos de biópsia foram obtidos de todas as cinco regiões geográficas do Brasil, corroborando o alcance nacional do registro, ainda que se reconheça que a predominância da região Sudeste possa influenciar a validade externa.

É necessário reconhecer algumas limitações deste estudo. A participação no registro continua sendo voluntária, com representação desigual entre as regiões, e a ausência de acompanhamento longitudinal limita, atualmente, as análises prognósticas. Além disso, a variabilidade no acesso a ferramentas diagnósticas avançadas, como a microscopia eletrônica e as técnicas moleculares, pode influenciar a classificação diagnóstica em um subconjunto de casos^39,40,41^. No entanto, o RBBR constitui o esforço nacional mais abrangente, até o momento, para caracterizar sistematicamente os achados das biópsias renais no Brasil.

Em conclusão, os dados de 2022 consolidam o RBBR como um registro nacional robusto e internamente consistente. A estabilidade dos padrões diagnósticos ao longo de anos consecutivos, aliada a um número crescente de biópsias registradas, corrobora a confiabilidade da plataforma e seu potencial para análises futuras. A expansão contínua do registro, juntamente com a incorporação de dados clínicos longitudinais, pode fornecer uma base valiosa para pesquisas epidemiológicas, para o planejamento de políticas de saúde e a implementação racional de terapias emergentes na nefrologia brasileira, bem como para apoiar o desenvolvimento de uma rede nacional de patologia renal mais integrada no país.

## Data Availability

Os conjuntos de dados gerados e/ou analisados durante o estudo estão disponíveis junto à Sociedade Brasileira de Nefrologia, mediante solicitação justificada.

## MATERIAL SUPLEMENTAR

O seguinte material online está disponível para o presente artigo:

Material Suplementar - List of collaborators.
